# MEDI3039, a novel highly potent tumor necrosis factor (TNF)-related apoptosis-inducing ligand (TRAIL) receptor 2 agonist, causes regression of orthotopic tumors and inhibits outgrowth of metastatic triple-negative breast cancer

**DOI:** 10.1186/s13058-019-1116-1

**Published:** 2019-02-18

**Authors:** Yoshimi Endo Greer, Samuel F. Gilbert, Brunilde Gril, Rajesh Narwal, Danielle L. Peacock Brooks, David A. Tice, Patricia S. Steeg, Stanley Lipkowitz

**Affiliations:** 1 Women’s Malignancies Branch, Center for Cancer Research, National Cancer Institute, Building 10, Room 4B54, Bethesda, MD 20892-1361 USA; 2 MedImmune, LLC, Gaithersburg, MD USA

**Keywords:** TRAIL, Death receptor, Agonist, Apoptosis, Breast cancer, Triple-negative breast cancer

## Abstract

**Background:**

TNF-related apoptosis-inducing ligand (TRAIL) receptor agonists are attractive anti-tumor agents because of their capability to induce apoptosis in cancer cells by activating death receptors (DR) 4 and 5 with little toxicity against normal cells. Despite an attractive mechanism of action, previous clinical efforts to use TRAIL receptor agonists have been unsuccessful. In this study, we examined MEDI3039, a highly potent multivalent DR5 agonist, in breast cancer cell lines and in vivo models.

**Methods:**

As in vitro model systems, we used 19 breast cancer cell lines that are categorized into four subtypes: ER+, HER2 amplified, basal A (triple-negative breast cancer) TNBC, and basal B TNBC. Cell viability was analyzed by MTS and RealTime live/dead assays. As in vivo model systems, MDA-MB231T orthotopic primary tumor growth in the mammary fat pad (MFP) and two experimental lung metastasis models were used. The effect of MEDI3039 on MFP tumors was assessed with immunohistochemical analysis. Lung metastases were analyzed with Bouin’s and H&E staining.

**Results:**

MEDI3039 killed multiple breast cancer cell lines, but the sensitivity varied among different subtypes. Sensitivity was basal B TNBC >> basal A TNBC > HER2 amplified > ER+ (average IC_50_ = 1.4, 203, 314, 403 pM, respectively). While the pattern of relative sensitivity was similar to GST-TRAIL in most cell lines, MEDI3039 was at least two orders of magnitude more potent compared with GST-TRAIL. In the MFP model, weekly treatment with 0.1 or 0.3 mg/kg MEDI3039 for 5 weeks inhibited tumor growth by 99.05% or 100% (median), respectively, compared with the control group, and extended animal survival (*p* = 0.08 or *p* = 0.0032 at 0.1 or 0.3 mg/kg, respectively). MEDI3039-induced caspase activation was confirmed in tumors grown in MFP (*p* < 0.05). In an experimental pulmonary metastasis model, MEDI3039 significantly suppressed outgrowth of surface (*p* < 0.0001) and microscopic metastases (*p* < 0.05). In an established lung metastasis model, MEDI3039 significantly inhibited growth of metastases (*p* < 0.01 in surface [> 4 mm], *p* < 0.01 in tumor percentage) and extended animal survival (*p* < 0.0001).

**Conclusion:**

MEDI3039 is a potent DR5 agonist in breast cancer cells in vitro and in vivo and has potential as a cancer drug in breast cancer patients, especially those with basal B TNBC.

**Electronic supplementary material:**

The online version of this article (10.1186/s13058-019-1116-1) contains supplementary material, which is available to authorized users.

## Background

Breast cancer is a heterogeneous group of diseases that may be stratified into subtypes based on the presence of distinct molecular markers [1]. Approximately 60–70% of breast cancers are estrogen receptor (ER) or progesterone receptor (PR) positive, and 15–30% of cases have gene amplification and overexpression of the human epidermal growth factor receptor 2 (HER2) protein [2]. Additionally, 10–15% of breast cancers are called “triple negative” (TNBC) defined by the lack of ER and PR expression and *HER2* amplification [2]. Patients with TNBC are commonly young (age < 50 years), disproportionately African–American, and the clinical course is frequently characterized by early relapse and poor overall survival [3]. Unlike the molecularly targeted treatment strategies available for hormone receptor expressing or *HER2* amplified subsets of breast cancer, effective targeted therapies for TNBC that improve survival have yet to be developed, and cytotoxic chemotherapy remains the main therapy for TNBC [4]. There is a clear need to develop effective, targeted therapies for TNBC.

Extensive characterization has revealed remarkable diversity in the molecular attributes of TNBC [5–8]. The majority of TNBC are basal-like, which is characterized by elevated expression of keratins 5/6 and 17, *TP53* mutation, aberrations in DNA repair pathways (e.g., *BRCA1* loss), and pro-proliferative gene expression [5]. Pre-clinically, basal-like TNBC cell lines have been further divided into basal A (epithelial) and basal B (mesenchymal) subtypes [7]. While the basal A subtype retains a more epithelial phenotype, the basal B subtype possesses mesenchymal and stem cell-like characteristics.

Previously, we reported that basal B subtype TNBC cell lines were effectively killed by glutathione-S-transferase (GST)-tagged tumor necrosis factor (TNF)-related apoptosis-inducing ligand (TRAIL), while cell lines representative of the other subtypes of breast cancer remained comparatively resistant [9]. TRAIL is a favorable therapeutic candidate in cancer treatment, because it activates the extrinsic death pathway with little normal cell cytotoxicity in vitro and in vivo [10]. TRAIL induces apoptosis via ligand binding to the death receptors DR4 and DR5, which triggers formation of the death-inducing signaling complex and recruitment and activation of caspase-8 [11], resulting in apoptotic cell death.

Recombinant human TRAIL (rhApo2L/TRAIL; dulanermin) and death receptor (DR) agonistic antibodies have been tested in a number of phase 1 and 2 clinical trials. These agents have established the safety of the approach and showed some anti-tumor activity; however, they were generally ineffective in improving patient outcomes (reviewed in [12, 13]), raising questions about the pharmacokinetic and pharmacodynamic properties. Dulanermin exhibited a short serum half-life (30 to 60 min in humans) [14]. DR agonistic antibodies have much longer half-lives (6–21 days) [15]; however, many of them require additional cross-linking to achieve optimal activity in vitro and are less potent as inducers of cell death compared with rhTRAIL ([16–18]). Reliable pharmacodynamic markers also need to be established for selecting dose and indication, monitoring of drug efficacy, and prediction of clinical outcomes [19, 20]. A study reported transient increases in apoptotic markers in mouse sera 8–24 h after treatment with dulanermin [21], providing proof of concept that apoptosis markers can be detected in patient serum. Stratification of patients based on predictive biomarkers may further help patients to benefit from the DR agonist therapy.

Recently, MEDI3039, a multivalent TRAIL receptor 2/DR5 agonist, has been developed to attempt to improve activity [22–24]. This multivalent protein is a modified protein derived from the third fibronectin type III domain of the glycoprotein tenascin C [23, 25], which possesses a region similar to the variable region characteristic of antibodies. An optimized multivalent DR5 agonist was highly potent in triggering cell death in multiple TRAIL-sensitive cell lines, was one to two orders of magnitude more potent than TRAIL, and showed promising results in multiple cancer cells (colon, lung, leukemia, liver cancers) and in vivo colon cancer models [23]. This study suggested that the multivalent DR5 agonist might have superior clinical activity in settings insensitive to the current therapeutic agonists that target this pathway.

In the present study, we tested MEDI3039 in 19 breast cancer cell lines and in vivo TNBC breast cancer xenografts. MEDI3039 was especially effective in cell lines representing the basal B TNBC subtypes and was at least two orders of magnitude more potent than GST-TRAIL. MEDI3039 significantly inhibited tumor growth and improved animal survival in mammary fat pad models. Moreover, MEDI3039 was remarkably effective in inhibiting lung metastasis outgrowth and extending animal survival, including in an established metastasis model. Herein, we report MEDI3039 as a promising and potent TRAIL receptor agonist for treatment of breast cancer patients, especially those with the basal B TNBC subtype.

## Methods and materials

### Cell lines

The HCC1937 and BT20 cell lines were obtained from Reinhard Ebner (Avalon Pharmaceuticals, Germantown, MD). The T47D, MCF7, BT474, MDA-MB453, SKBR3, HCC1954, AU565, HCC1599, HCC1187, MDA-MB468, MDA-MB231 (MB231), Hs578t, MDA-MB436, BT549, and HCC38 cell lines were obtained from American Type Culture Collection (Manassas, VA, USA). ZR75-1 and HCC1500 were from the Steeg laboratory. All cancer cell lines were grown in RPMI 1640 base media (Themo Fisher Scientific, Waltham, MA) supplemented with 10% fetal calf serum (FBS), 100 units/mL of penicillin, and 100 units/mL of streptomycin (P/S). Authenticity of the cell lines was verified by PowerPlex 16 HS System at Laragen Inc. (Culver City, CA). Cell identification was performed multiple times (Jan. 29, 2015; Jul. 30, 2015; Mar. 6, 2017). A subline of human MB231 cells, designated MDA-MB-231 T (MB231T) [26], was generously provided by Dr. Zach Howard (Laboratory of Immunoregulation, National Cancer Institute, Bethesda, MD) and was maintained in DMEM (cat#11965092, Thermo Fisher Scientific) supplemented with 10% FBS. The MB231T cell line was used for its reliable in vivo experimental metastatic potential. The MB231T was confirmed as a subline of MB231 cells by PowerPlex 16 HS System at Laragen Inc. (May 22, 2018). Primary human foreskin fibroblasts (HFF) [27] were kindly provided by Dr. Kenneth Yamada (National Institute of Dental and Craniofacial Research, NIH) and were maintained in DMEM supplemented with 10% FBS and 100 units/mL of P/S at 37 °C, 10% CO_2_ incubator.

### Chemicals

Z-VAD-FMK, a pan-caspase inhibitor (cat# 2163, R&D Systems, Inc. Minneapolis, MN), was reconstituted in dimethyl sulfoxide (DMSO) at 10 mM and stored at − 30 °C. GST-TRAIL was prepared in the laboratory as previously reported [9].

### MEDI3039

MEDI3039 was provided by MedImmune (Gaithersburg, MD) and was stored in liquid form at a concentration of 10.2 mg/mL in its formulation buffer (10 mM NaPhosphate, 250 mM Trehalose, 0.02% PS-80, pH = 6.0) in the dark at 4 °C. For in vitro experiments, MEDI3039 was diluted into RPMI1640 cell culture medium at 10^−6^ M and used at concentration ranging from 10^−19^ to 10^−9^ M. For in vivo experiments, the stock solution of MEDI3039 (mentioned above) was diluted into sterile Ca^2+^/Mg^2+^-free phosphate-buffered saline (PBS) at the final concentrations of 0.003, 0.01, and 0.03 mg/mL, for 0.03, 0.1, and 0.3 mg/kg each treatment group.

### Cell viability assay (MTS assay)

Cells were plated in clear 96-well plates at a density of 5000 cells/well, then treated under the experimental conditions described in the body of the text. Viability was subsequently determined using the CellTiter 96® AQueous One Solution Cell Proliferation Assay (cat# G3582, Promega Corporation, Madison, WI) as previously described [9]. At least three independent experiments were carried out per cell line, with three replicates per experiment. Results were shown as the mean ± SEM of at least three independent experiments.

### RealTime live/dead assay

Cells were plated in black 96-well plates at a density of 1250 cells/well. Next day, media was replaced with MEDI3039 or GST-TRAIL-containing media supplemented with RealTime-Glo™ MT Cell Viability Assay reagent (cat#G9712, Promega) and CellTox™ Green Cytotoxicity Assay reagent (cat#G8743, Promega). Live or dead status was monitored by luminescence or fluorescence (Ex485/Em525) measurement, respectively, up to 72 h, according to the manufacturer’s protocols.

### Caspase-3/7 activity assay

Cells were treated with MEDI3039 or GST-TRAIL as indicated in the text and figure legends. Caspase activity was assessed using the Caspase-Glo® 3/7 assay system (cat# G8093, Promega Corporation, Madison, WI) as previously described [28]. Three independent experiments were performed and normalized to the control cells. The results were shown as mean ± SEM.

### siRNA transfection experiments

Small interference RNAs for DR4 (DR4_#1 [cat#SI00056728] and DR4_#3 [cat# SI00056742]) or DR5 (DR5_#1 [cat # SI00056700] and DR5_#6 [SI03094063]) and AllStars Negative control (cat # SI03650318) were obtained from Qiagen (Valencia, CA). MB231 cells were plated on 10-cm tissue culture dishes and grown in RPMI supplemented with 10% FBS for 48 h prior to transfection. Reverse transfections were performed by incubating siRNA at a concentration of 50 nM in 3 mL of Opti-MEM (Thermo Fisher Scientific) with 15 μl of Lipofectamine RNAiMax transfection reagent (cat # 13778-150, Thermo Fisher Scientific). After 20-min incubation at room temperature (RT) for complex formation between the RNAiMax and the siRNA, cells in suspension (3.0 mL at 1.0 × 10^5^ cells/mL in RPMI supplemented with 10% FBS) were added to the siRNA mix and incubated for 5 min. Then, cells were distributed to 96-well plates with a final cell concentration of 5000 cells/well for MTS assays, and 6-well plates at 500,000 cells/well for Western blotting. After 48 h, cells were treated with either MEDI3039 or GST-TRAIL at indicated concentration and incubated for 3 days. Expression of DR4 and DR5 were measured at 96 h post transfection.

### Western blotting

Eighty to 90% confluent monolayers that had been seeded in 6- or 12-well cell culture plates were rinsed twice with PBS, lysed, and processed for SDS–PAGE and Western blot analysis as previously described [9]. After proteins were separated by sodium dodecyl sulfate polyacrylamide gel electrophoresis and transferred to polyvinylidene difluoride membranes, the membranes were probed with antibodies and then visualized on Odyssey Fc imager (LI-COR, Lincoln, NE), using Image Studio software. The following antibodies were used: anti-DR4 (cat#GTX28414, GeneTex, Irvine, CA), anti-DR5 (cat#sc-65314, Santa Cruz, Dallas, TX), and anti-HSC70 (cat#sc-7298, Santa Cruz). Caspase-8 (#9346), cleaved caspase-8 (#9496), caspase-3 (#9662), cleaved caspase-3 (#9661), PARP (#9542), cleaved PARP (#5625), ERα (#8644), HER2 (#4290), Axl (#8661), and EGFR (#2232) were from Cell Signaling (Danvers, MA). Vimentin (#550513) was from BD Biosciences (San Jose, CA).

### MEDI3039 pharmacokinetics (PK)

To determine the half-life of MEDI3039, drug was injected at 6.5 mg/kg by *i.v.* into 6–8-week-old Colo-205 bearing female athymic nu/nu mice. Different groups of mice were bled 100 μL per time point at 5 min, 15 min, 30 min, 1 h, 2 h, 4 h, 8 h, 16 h, 24 h, 48 h, 72 h, 96 h, or 120 h. Serum concentrations of drug were measured by ELISA by coating plates with recombinant TRAIL-R2 and incubated with serum diluted in PBS-T containing 1% milk. Dilutions were adjusted for each time point in order for the signal to be within the dynamic range of the assay. Bound drug was detected with a 1 in 10,000 dilutions of polyclonal anti-Tn3 rabbit serum (Covance, Princeton, NJ) in PBS-T containing 1% milk followed by a 1 in 10,000 dilutions of donkey anti-rabbit HRP (Jackson ImmunoResearch, West Grove, PA). A standard curve was generated for each construct.

### In vivo animal studies

All mouse experiments were performed under an approved National Cancer Institute Animal Use Agreement. In all experiments, female 5-week-old athymic NRC nu/nu mice were used.

#### Animal model 1: mouse mammary fat pad (MFP) xenograft model

This experiment was repeated twice. The first experiment is summarized in Fig. 4; the second experiment is in an Additional file.

##### Study design

To investigate the effect of MEDI3039 on primary tumor growth, 5 × 10^6^ MB231T human breast cancer cells/50 μl in sterile medium were implanted into the #4 and/or #9 mammary fat pad (MFP) of mice. Once the average tumor size reached 200 mm^3^, mice were randomized into treatment groups. In the first experiment, mice were assigned to two treatment groups: vehicle or 0.3 mg/kg MEDI3039, weekly, for 2 weeks. Drug was administered via tail vein injection, 0.25 mL/25 g body weight. In the second experiment, mice were assigned to one of the four treatment groups: vehicle, 0.01, 0.1, or 0.3 mg/kg MEDI3039, and drug was administered weekly, for 5 weeks via tail vein injection, 0.25 mL/25 g body weight. Tail vein injection is only practical up to five times; therefore, we did not continue injections beyond five doses. In order to verify the effect of MEDI3039 on apoptosis in tumors, 5 out of 15 mice were sacrificed 16–20 h after the first drug treatment. The growth of the primary tumor in the MFP and body weight were monitored twice weekly. Once tumor size reached 20 mm in a single dimension, animals were euthanized. MFP tumors were dissected into two parts, and one was fixed-frozen as follows: incubation in 4% paraformaldehyde for 24 h at 4 °C, followed by incubation in a 20% sucrose solution overnight at 4 °C, and embedded in optimal cutting temperature (OCT) compound, and dry ice bath. The other half was fixed with 10% NBF, 24 h, and then switched to 70% EtOH for paraffin embedding.

##### Tissue collection and immunofluorescence staining

Tumor samples embedded in OCT were sectioned at 5 μm using CryoStar (Thermo Fisher) and placed on microscope slides (Superfrost Plus, cat#12-550-15, Fisher Scientific, Pittsburgh, PA). Slides stored at − 80 °C were equilibrated on dry ice for 20 min, washed in 4 °C PBS for 5 min to remove excess OCT, then placed into cold methanol for 5 min at − 20 °C. After washing with PBS three times, 5 min each, tissue samples were pre-blocked with 5% goat serum (cat# X090710 [Dako] or cat#G9023 [Sigma-Aldrich]) in PBS at RT for 20 min. Anti-cleaved-caspase-3 (cat#9661, Cell Signaling) and anti-Ki-67(cat # VPRM04 [Vector laboratories] or cat#SAB5500134 [Sigma-Aldrich, St. Louis, MO]) were diluted at 1:200 with 5% goat serum/PBS, then the diluted antibody solution was placed on top of the tissue section circled with PAP pen (cat#Z672548, Sigma). Tissue slides were kept in a humidified chamber (with wet paper) in a cold room 4 °C overnight. Next day, primary antibody solutions were removed and slides were rinsed with PBS three times, 5 min each. Secondary antibody Alexa Fluor goat-anti-rabbit 488 (cat#A-11008, Thermo Fisher Scientific) was diluted at 1:500 with 5% goat serum and combined with 4′,6-diamidino-2-phenylindole dihydrochloride (DAPI) (1200 dilution of 5 mg/mL stock, cat#32670, Sigma-Aldrich), and 150 μl of the mixture of the second antibody and DAPI was placed on each tissue sample. Tissues were incubated 1 h, at RT in the dark. After washing with PBS for three times, 5 min each, slides were quickly dipped into ddH_2_O, and 1 drop of mounting medium (cat# GM30411-2 [Dako] or cat#P36930 [Thermo Fisher Scientific]) was placed on top of each tissue sample. A coverslip was placed, and slides were dried at least 20 min before taking images.

##### Image analysis of immunostaining experiments

One section per mouse (*n* = 5 per treatment group) was analyzed. For each section, three pictures of “hot spots” (hot spots are defined as the region of the section with the highest staining for Ki67 or cleaved caspase-3) were acquired at × 100 magnification using Zeiss Axioskop. A previous pilot analysis confirmed that quantification of three hot spots per section was representative of the whole section as it gave similar results compared to the quantification of the entire section. Zen© software was used to calculate percent area stained by each immunofluorescence marker (Ki67 and cleaved caspase-3), as well as DAPI. The area stained by either Ki67 and cleaved caspase-3 was then normalized to the percent area stained by DAPI.

##### Pathological analysis of extra mammary fat pad tumor

During the second MFP experiment, one mouse treated with 0.3 mg/kg MEDI3039 developed a tumor outside of the MFP. In order to identify the origin of the tumor, when size reached > 20 mm in diameter, the tumor was excised and formalin-fixed, paraffin embedded. The tumor sample was immunohistochemically analyzed at Pathology/Histotechnology Laboratory, Laboratory Animal Sciences Program, NCI-Frederick. Human-specific anti-mitochondrial antibody [MTC02] (ab79479, abcam, Cambridge, UK) was used to diagnose the species of origin of the tumor.

#### Animal model 2: an experimental pulmonary metastasis mouse model

To investigate the effect of MEDI3039 on the outgrowth of metastases of human breast cancer cells in vivo, an experimental pulmonary metastasis study was conducted. This experiment was repeated twice. The first experiment is shown in an Additional file 4, and the second experiment is shown in Fig. 5. Mice were injected with 7.5 × 10^5^ MB231T cells/50 μl in Ca^2+^/Mg^2+^ free-PBS via the tail vein [26]. On the day following injection (day 1), mice were randomized into treatment groups containing 15 mice each. In the first experiment, the mice were assigned to one of the following three treatment groups: vehicle, 0.3 mg/kg, and 1.0 mg/kg MEDI3039, and in the second experiment (Fig. 5), the mice were assigned to one of the following two treatment groups: vehicle or 0.3 mg/kg MEDI3039. Vehicle or MEDI3039 were given on days 4, 7, 11, and 14 via tail vein. Mice were sacrificed at day 54 (in the first experiment) or day 91 (in the second experiment), and the lungs from 10 mice per group were inflated and fixed in Bouins’ solution. Surface metastatic lesions were counted on all lungs in Bouins’ fixative using a magnifying glass. The lungs from 5 mice per group were examined with hematoxylin and eosin (H&E) stained, formalin-fixed paraffin-embedded (FFPE) step sections for histologic analysis [26]. Five-level sections of H&E-stained lung tissue slides were scanned using a Zeiss Axioskop microscope, and images were captured with Axiocam 506 Color.

#### Animal model 3: established pulmonary metastasis mouse model (Fig. 6)

The therapeutic effect of MEDI3039 on established pulmonary metastasis of human breast cancer cells was examined. A total of 40 mice were injected with 7.5 × 10^5^ MB231T cells/50 μl in Ca^2+^/Mg^2+^ free-PBS via the tail vein [26]. We planned to euthanize 2 mice on days 20, 27, 34, 41, and 48, to determine if lung metastasis were established and subsequently start drug treatment in the rest of the mice. In our experiment, lung surface metastases were confirmed in both mice sacrificed on day 20 by examining Bouin’s fixed lung tissues. Subsequently, the remaining animals were randomly divided to vehicle (*n* = 19) and MEDI3039 (*n* = 19) treatment groups, and vehicle or MEDI3039 (0.3 mg/kg) was given on days 23, 27, 30, and 34 via tail vein. The mice were followed up until day 90. Mice were sacrificed when they became moribund. At the time of necropsy, lung tissues were collected; the lungs from 14 animals per group were fixed in Bouin’s solution and analyzed for surface metastases, and the lungs from 5 animals per group were fixed for FFPE and examined for histologic analysis by H&E staining. In the gross examination, the numbers of tumors were counted using a dissecting microscope in all lobes, blinded as to group. The number of tumors was classified according to their size (≤ 1 mm, 2–3 mm, 4–5 mm, > 5 mm). In histological analysis, three-level sections of H&E-stained lung tissue slides were scanned using Aperio AT2 scanner (Leica Biosystems, Buffalo Grove, IL) into whole slide digital images. All image analysis was performed using Halo imaging analysis software (Indica Labs, Corrales, NM), and image annotations were performed by one pathologist (B.O.K) who was blinded to the status of the groups of mice. The lung lobes were annotated including areas of invasive tumor. Surrounding tissues such as the diaphragm and lymph nodes were excluded from the analysis. Areas of necrosis and inflammation were also ignored and were not included in the determination of percent malignant tumor cells. Random forest tissue classifier [29, 30] was used to automatically differentiate tumor cells from normal lung parenchyma.

### Sensitivity analysis

The sensitivity plot was made by plotting the IC_50_ for both MEDI3039 and GST-TRAIL in a log scale scatter-plot using R project (https://www.r-project.org/). IC_50_ > 1000 and > 100,000 were set as 1000 and 100,000, respectively.

### Statistical analysis

**Fig. 1:**
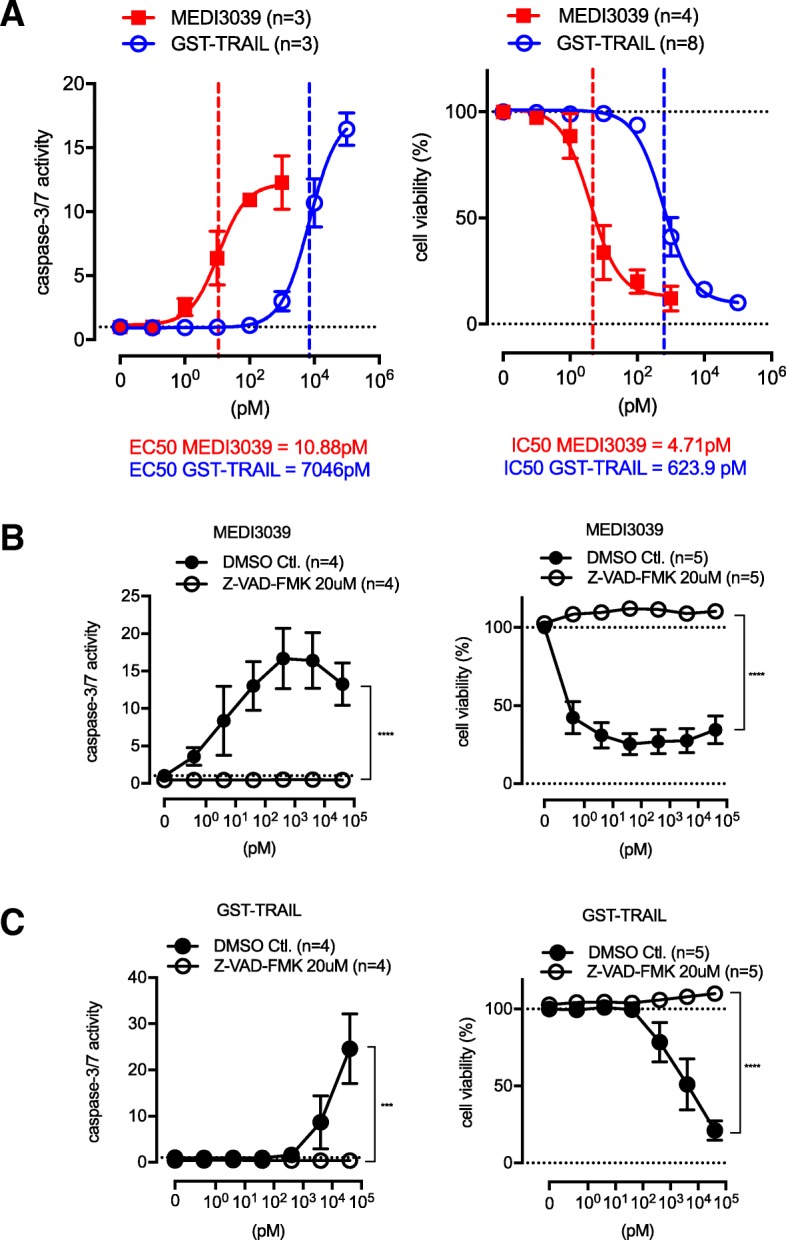
MEDI3039 induces apoptotic cell death in basal B TNBC MB231 cells. **a** MEDI3039-induced caspase activation (left) and cell death (right) in MB231 cells. MEDI3039 was at least two orders of magnitude more potent compared with GST-TRAIL. MB231 cells were treated with drugs for 1 h for caspase assays and 72 h for MTS assays. Data is shown as average ± SEM of multiple independent experiments. EC_50_ and IC_50_ were obtained by GraphPad Prism Nonlinear regression analysis. **b** Z-VAD-FMK, a pan caspase inhibitor, completely blocked caspase-3/7 activation (left) and cell killing effect (right) of MEDI3039. Cells were pretreated with Z-VAD-FMK for 60 min before treated with MEDI3039. Data is shown as average ± SEM of multiple independent experiments. ****p < 0.0001, two-way ANOVA. **c** Z-VAD-FMK completely blocked caspase-3/7 activation (left) and cell killing effect (right) of GST-TRAIL. Data is shown as average ± SEM of multiple independent experiments. ***p < 0.001, ****p < 0.0001, two-way ANOVA

**Fig. 2:**
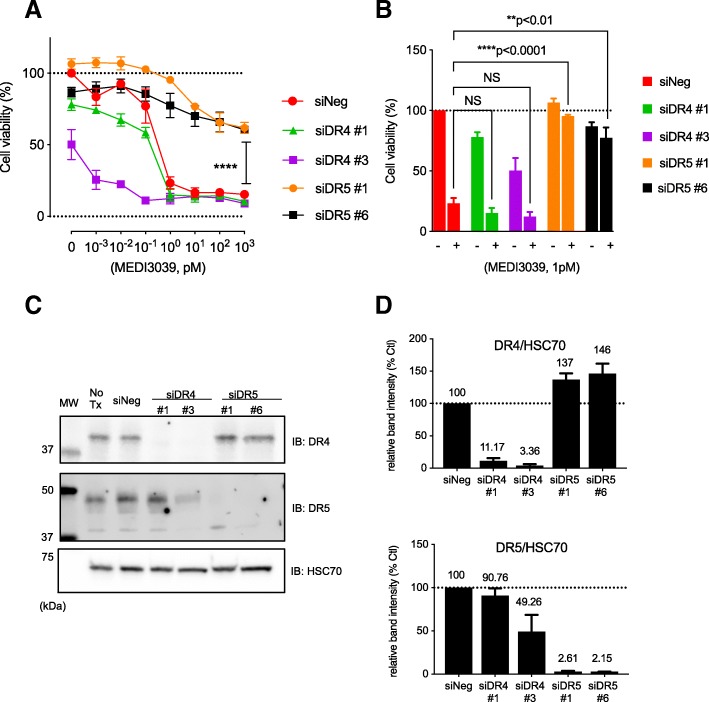
MEDI3039-dependent cell death is mediated by DR5. **a** MB231 cells were transfected with Neg Ctl. siRNA (siNeg), two separate DR4 siRNAs (siDR4 #1 and #3), or two separate DR5 siRNAs (siDR5 #1 and #6). Two days later, MEDI3039 was added and incubated for an additional 3 days. Cell viability was examined by MTS assays. Data is shown as average ± SEM of three independent experiments. ****p < 0.0001, two-way ANOVA, siNeg vs siDR5_#1, siNeg vs siDR5_#6. **b** The effect of MEDI3039 at 1 pM was compared among siNeg, siDR4, and siDR5. Statistical analysis was performed with Student’s *t* test. **c** Representative western blot showing effective knockdown of endogenous DR4 and DR5 proteins by corresponding siRNAs. HSC70 was used as a loading control. **d** Quantitative analysis of band intensities in western blot analyses. Data is shown as mean ± SEM of three independent experiments, and band intensities are shown as % relative to siNeg ctl

A variety of two-way analyses of variances (ANOVA) was used in Figs. 1b, c and 2a and Additional file 1B, C, and the differences were considered significant when the *p* value was less than 0.05. Student’s *t* test was used for Figs. 2b and 6c. A non-parametric test using the Mann–Whitney test was used to compare distributions if the data were not normally distributed and/or the sample sizes were less than 10 mice per group (e.g., Figs. 4c, 5e, and 6e). Unpaired *t* test was used to compare the number of metastasized tumors in two groups with a sample size of 10 each (Fig. 5c). One-way ANOVA was performed on data presented in Fig. 4d bottom. Actuarial analyses were performed on the survival data using the Kaplan–Meier method, and curves were analyzed with log-rank (Mantel–Cox) test (e.g., Figs. 4f, 5d, and 6a). Survival times were censored if the mouse was alive at the time of the last follow-up. All *p* values were two-sided.

## Results

### MEDI3039 induced cell death via caspase activation at lower concentrations compared with GST-TRAIL in MB231 cells

First, the effect of MEDI3039 on caspase activity was tested and compared with that of GST-TRAIL in MB231 cells. MEDI3039 stimulated caspase-3/7 activation at lower doses (> 1 pM) compared to GST-TRAIL (> 1 nM) (Fig. 1a, left panel) after a 1-h treatment. The EC_50_ for MEDI3039-induced caspase activation (10.88 pM) was ~ 600-fold lower than the EC_50_ for GST-TRAIL (7046 pM). A time-dependent effect of caspase activation induced by MEDI3039 was also observed by Western blotting. MEDI3039 stimulated cleavage of caspase-8, 3, and PARP as early as 1 h in MB231 cells (Additional file 1A). Next, the effect of MEDI3039 on cell viability was tested with MTS assays after 3 days of treatment. MEDI3039 induced cell death at a significantly lower concentration (IC_50_ = 4.71 pM) compared with GST-TRAIL (IC_50_ = 623.9 pM) in MB231 cells (Fig. 1a, right panel). MEDI3039-dependent caspase activation and loss of viability were completely abrogated when cells were pretreated with the pan-caspase inhibitor Z-VAD-FMK for 60 min (Fig. 1b). Similar results were obtained with GST-TRAIL (Fig. 1c).

The drug’s effect was also compared at 16 h and 72 h treatment. As shown in Additional file 1B, the IC_50_ of MEDI3039 was decreased from 8.5 to 1.3 pM in MB231 cells and from 6.5 to 0.99 pM in MB468 cell lines with longer treatment. As MEDI3039 has a half-life of 15 h in mice and 31 h in monkeys (see Table 1 below and the “Discussion” section), tumors in animals would be exposed to the drug for a longer period of time. Therefore, we used a 72-h treatment for further experiments. We also tested the drug effect on non-malignant, HFF cells. As shown in Additional file 1C left panel, HFF cells were resistant to MEDI3039 compared to MB231 cells. Both DR4 and DR5 were expressed in HFF (Additional file 1C right panel), although the expression levels were less compared with MB231. Prior work from our lab has shown that the level of DR does not correlate with the response to TRAIL receptor agonists [9].

**Table 1:** Pharmacokinetics of MEDI3039 in mice

	Half-life	Tmax	Cmax	Clast	AUCall	AUCINF_obs	CL	Vss
	(h)	(h)	(ug/mL)	(ug/mL)	(ug h/mL)	(ug h/mL)	(mL/h/kg)	(mL/kg)
MEDI3039	15.4	0.25	130.76	0.06	828.9	830.84	7.82	79.71

To confirm that MEDI3039 and GST-TRAIL are inducing cell death, we monitored live and dead cells with RealTime Glo™ Cell Viability Assay and CellTox™ Green Cytotoxicity Assay (Additional file 1D, E). Both MB231 and MB468, TRAIL-sensitive cell lines, showed a dose-dependent induction of cell death and decrease of live cells by both MEDI3039 and GST-TRAIL. The cell death of MB231 reached a maximum level at 24 h, whereas MB468 did so at 48–72 h (Additional file 1E). In contrast, MEDI3039 and GST-TRAIL induced little death in MCF7 and T47D with up to 72 h of treatment (Additional file 1E).

### MEDI3039-induced cell death is mediated by DR5

MEDI3039 was developed as a multivalent agonist against TRAIL-R2/DR5 [23]. To verify the mechanism of action of MEDI3039, endogenous DR4 and DR5 expression was decreased by siRNA transfection. In siRNA negative control (siNeg)-transfected MB231 cells, MEDI3039 inhibited cell viability in a dose-dependent manner. The IC_50_ of MEDI3039 in siNEG-transfected MB231 cells was 0.5–1 pM (Fig. 2a) while it was 4.71 pM in the untransfected MB231 cells in Fig. 1a. We attribute the decrease of IC_50_ in the siRNA-transfected cells in part to siRNA transfection prior to 3 days of MEDI3039 treatment. This cell killing by MEDI3039 was not reversed with either of the two DR4 siRNAs. On the other hand, MB231 cells transfected with DR5 siRNAs were resistant to MEDI3039 (Fig. 2a, b). These data support that MEDI3039 is a DR5-specific TRAIL receptor agonist. Western blot confirmed that endogenous DR4 or DR5 were effectively decreased by siRNA transfection targeting each gene (Fig. 2c, d).

### TNBC were most sensitive to MEDI3039

**Fig. 3:**
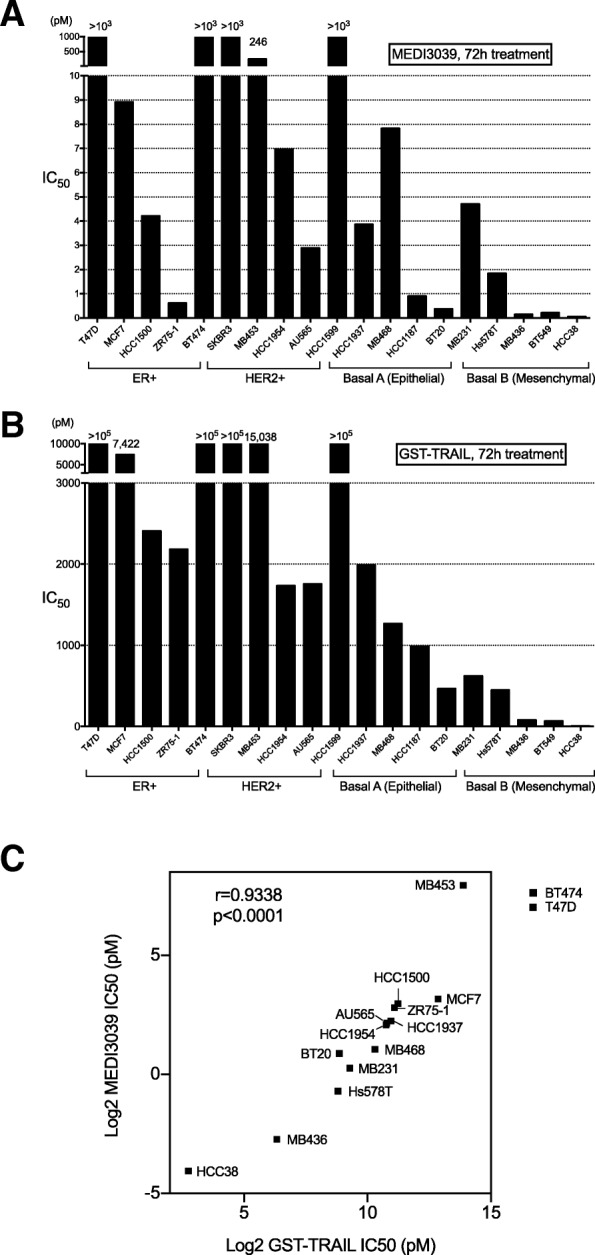
MEDI3039 sensitivity is different depending on breast cancer subtypes. IC_50_ of MEDI3039 (**a**) and IC_50_ of GST-TRAIL (**b**) were obtained by GraphPad Prism Nonlinear regression analysis of multiple MTS assays with 3 days of cell culture (at least three times per cell line). **c** Correlation of IC_50_ between MEDI3039 and GST-TRAIL in 15 breast cancer cell lines. Note that BT474 and T47D were out of scale because IC_50_ could not be determined due to resistance to MEDI3039 and GST-TRAIL

The effects of MEDI3039 and GST-TRAIL on viability were further examined in 19 breast cancer cell lines representing different subtypes of breast cancer (ER+, HER2 amplified, basal A TNBC, and basal B TNBC). Western blots of 15 of the cell lines showing different markers are shown in Additional file 2). MEDI3039-induced cell death was prominent in most TNBC, particularly basal B cell lines; however, there were a few non-TNBC cell lines that were sensitive to MEDI3039 (HCC1500, ZR-75-1, AU565, Fig. 3a). GST-TRAIL also induced cell death most effectively in TNBC cell lines, particularly basal B cell lines (MB231, Hs578T, MB436, BT549, and HCC38), and many of the ER+ and HER2 amplified cell lines exhibited resistance to GST-TRAIL (Fig. 3b), consistent with our previous report [9]. Sensitivity plot analysis showed a strong correlation between MEDI3039 and GST-TRAIL sensitivity across the cell lines (Fig. 3c). Comparison between the IC_50_ of MEDI3039 and those of GST-TRAIL indicates that MEDI3039 is approximately 100–1000 times more potent compared to GST-TRAIL at inducing cell death in the sensitive cell lines (Additional file 3).

### Pharmacokinetics of MEDI3039

Pharmacokinetic parameters for MEDI3039 measured in 6–8-week-old Colo-205 bearing female athymic nu/nu mice dosed at 6.5 mg/kg showed a half-life of 15.4 h and a Cmax 130.76 μg/mL (1.59 μM), AUC 828.9 (μg h/mL) (Table 1). The Cmax at this dose (~ 1.5 μM) was substantially higher than IC_50_ (picomolar range) for the sensitive breast cancer cells tested (Additional file 3).

### MEDI3039 inhibited tumor growth and improved survival in mouse mammary fat pad (MFP) xenografts

The effect of MEDI3039 on TNBC was tested in a mouse MFP xenograft model. In the first MFP experiment (Additional file 4), MB231T cells were injected into mouse MFP. Once the average size of the breast tumors reached 200 mm^3^ (on approximately day 18), vehicle (*n* = 15) or MEDI3039 (0.3 mg/kg, *n* = 15) were injected twice, 1 week apart, intravenously via tail vein. Five mice from each group were sacrificed 16–20 h after the first drug injection to examine the effect of drugs on tumor samples by immunohistochemistry. MEDI3039 significantly reduced the Ki67 signal and induced apoptotic marker cleaved-caspase-3 in tumor samples compared with the vehicle-treated group. The median for Ki67 normalized to DAPI area staining was 0.7601 (interquartile range [IQR] = 0.6556–1.004) in vehicle-treated mice, and it decreased by 62% with a median of 0.2859 (IQR = 0.21–0.5339; *p* = 0.0317) in MEDI3039-treated mice. Conversely, cleaved-caspase 3/ DAPI was significantly increased by 95% in MEDI3039-treated mice compared to vehicle-treated mice (vehicle median 0.02528, IQR = 0.0068–0.1332, MEDI3039 median = 0.4844, IQR = 0.3429–0.7443; *p* = 0.0079, Additional file 4B, C). After the second drug injection on day 25, 10 mice per each group were followed with measurement of body weight and tumor size. In the vehicle control group, the average size of tumors reached 20 mm in diameter on day 35, and all the mice in the group needed to be sacrificed. In contrast, significant tumor regression was observed in the MEDI3039-treated group until around day 50, although the tumors eventually regrew in all mice (Additional file 4D). Animals were followed up to day 92. MEDI3039 significantly (*p* < 0.0001, log-rank test) extended animal survival (Additional file 4E). The median survival for the MEDI3039-treated mice was 72 days, extending survival by 37 days. A hazard ratio of 0.3333 was obtained for 0.3 mg/kg MEDI3039 compared with the vehicle control, with 95% CI ratio of 0.1211–0.9172.

**Fig. 4:**
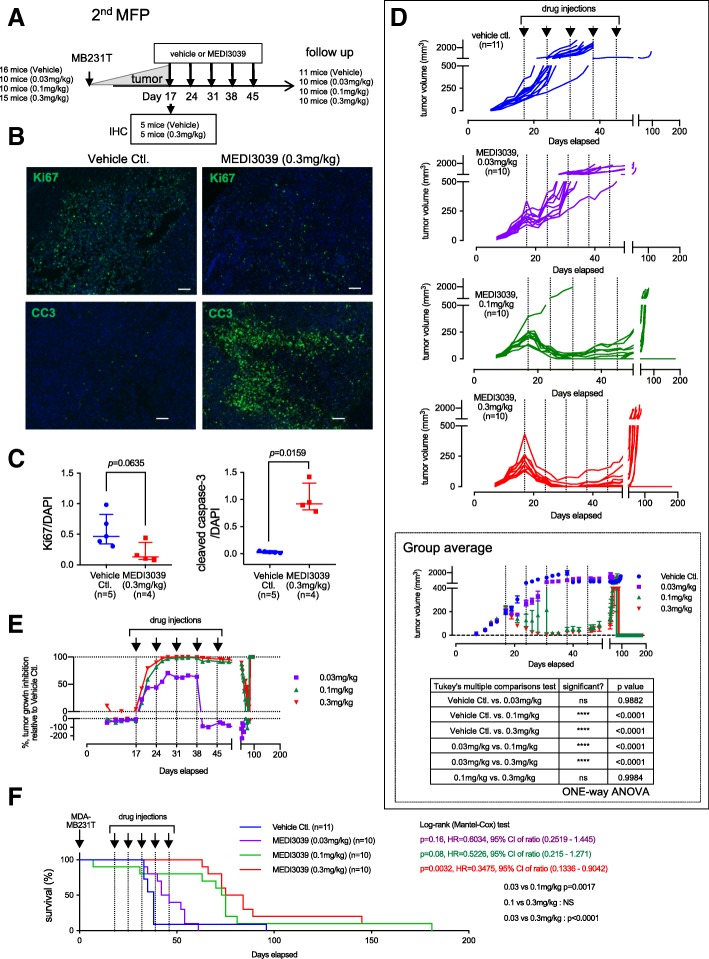
MEDI3039 inhibited tumor growth and extended animal survival in the MB231T mammary fad pad model. **a** Design of the experiment. MEDI3039 was administered weekly, for 5 weeks at indicated doses. Five mice in the control and 0.3 mg/kg group were sacrificed for histology analysis and 10 mice per group were followed for tumor growth and survival. **b** Immunohistochemistry analysis of tumor samples from MEDI3039 (0.3 mg/kg) or vehicle-injected mice. Samples were stained with either Ki67 and DAPI or CC3 (cleaved caspase 3) and DAPI. Bar = 100 μm. **c** Quantitative analysis of signal intensity of Ki67 and CC3, both normalized with DAPI. Data is shown as median with IQR. Numbers of mice examined was 5 (vehicle control.) and 4 (MEDI3039 0.3 mg/kg). *p* value was obtained by Mann–Whitney test. **d** Tumor growth curve in each treatment group. The bottom panel shows averages for all mice at each dose. One-way ANOVA was used to compare statistical significance between different groups. **e** Tumor growth inhibition curve. Data is shown as median % tumor inhibition of 0.03, 0.1, and 0.3 mg/kg MEDI3039 groups, compared with the vehicle control group. **f** Survival curve of mice treated with MEDI3039 at indicated doses. Median survival was 38 days (vehicle control group), 44 days (MEDI3039, 0.03 mg/kg group), 74 days (MEDI3039, 0.1 mg/kg group), 79.5 days (MEDI3039, 0.3 mg/kg group). *p* value, HR (hazard ratio), and 95% of CI (confidence interval) were obtained by log-rank (Mantel–Cox) test, compared with the vehicle control group

In the second MFP experiment (Fig. 4a), we tested MEDI3039 at multiple doses (0, 0.03, 0.1, 0.3 mg/kg, *n* = 16, 10, 10, 15 each) and more injections (weekly doses for 5 weeks) to evaluate dose-dependent effects and to test if more doses would prevent the relapses seen in the first experiment (Additional file 4). Five mice from the vehicle control and 0.3 mg/kg MEDI3039 groups each were sacrificed 16–20 h after drug injection to examine the effect of drugs on tumor samples by immunohistochemistry, and the rest of the mice were followed up to day 181. Similar to the first experiment, MEDI3039 (0.3 mg/kg) treatment induced a modest decrease of Ki67 intensity in tumor samples and increased cleaved caspase-3 (Fig. 4b, c). In vehicle-treated mice, the median for Ki67/DAPI area staining was 0.4693 (IQR = 0.3464–0.8281). In the MEDI3039-treated mice, the Ki67/DAPI area staining decreased by 73% with a median of 0.1308 (IQR = 0.0889–0.3691; *p* = 0.0635). The staining for cleaved-caspase 3/DAPI was significantly increased by 97% in MEDI3039-treated mice compared to vehicle-treated mice, vehicle median 0.02881, IQR = 0.01801–0.0472, and MEDI3039 median = 0.922, IQR = 0.8087–1.3; *p* = 0.0159. Rapid tumor growth was observed in the vehicle control group and only 1 mouse was alive at day 40 (Fig. 4d, top). In contrast, MEDI3039 showed dose-dependent inhibitory effects on tumor growth. At day 35, while the average size of tumors in the vehicle-treated group reached 1719 mm^3^ (*n* = 8), in the mice treated with 0.1 and 0.3 mg/kg, the average tumor size regressed to 23.86 mm^3^ (*n* = 9) and 9.72 mm^3^ (*n* = 10), respectively. The group average of tumor growth curve (Fig. 4d, bottom) showed that 0.03 mg/kg did not inhibit tumor growth effectively; however, 0.1 and 0.3 mg/kg MEDI3039 resulted in significant tumor regression. Tumor regression curves relative to tumor vehicle control illustrates immediate and lasting anti-tumor growth effect of the two higher dosages of MEDI3039 (Fig. 4e). At day 35 (after the third dosing of MEDI3039), tumor growth was almost completely inhibited (99% in 0.1 mg/kg, 100% in 0.3 mg/kg). The lowest dosage of MEDI3039 (0.03 mg/kg) showed only transient tumor regression. The primary tumor became undetectable in 1 out of 10 mice (0.1 mg/kg group) and 2 out of 10 mice (0.3 mg/kg group). Kaplan–Meier analysis of the mouse survival demonstrated a progressive increase in survival with increasing dosage of MEDI3039. Log-rank (Mantel–Cox) test showed that while 0.03 mg/kg MEDI3039 did not improve survival compared with the vehicle control (*p* = 0.16), 0.1 mg/kg and 0.3 mg/kg showed modest and significant improvement of animal survival (*p* = 0.08, *p* = 0.0032, respectively). Log-rank test between MEDI3039 groups showed that 0.1 mg/kg was more effective than 0.03 mg/kg (*p* = 0.0017) and 0.3 mg/kg was more effective than 0.03 mg/kg (*p* < 0.001); there was no statistical difference between 0.1 and 0.3 mg/kg. The hazard ratio of the survival for mice treated at 0.3 mg/kg MEDI3039 was 0.3475 compared with the vehicle control with 95% CI ratio of 0.1336–0.9042 (Fig. 4f). Median survival was 38 days with the vehicle control, 44 days at 0.03 mg/kg, 74 days at 0.1 mg/kg, and 79.5 days at 0.3 mg/kg MEDI3039.

One of the mice treated with 0.3 mg/kg MEDI3039 had a complete response in the tumor and remained tumor free until day 73. However, a small mass started to develop outside the MFP at day 75. The mouse was sacrificed on day 145 due to the size of this tumor > 20 mm in diameter (Additional file 5A). Pathological analysis confirmed that the extra-MFP tumor was human origin and poorly differentiated invasive carcinoma that stained positively with an anti-human mitochondrial antibody (Additional file 5B). Therefore, we conclude this tumor was a metastatic tumor from the MB231T cells originally injected in the MFP.

### MEDI3039 inhibited lung metastasis tumor outgrowth

**Fig. 5:**
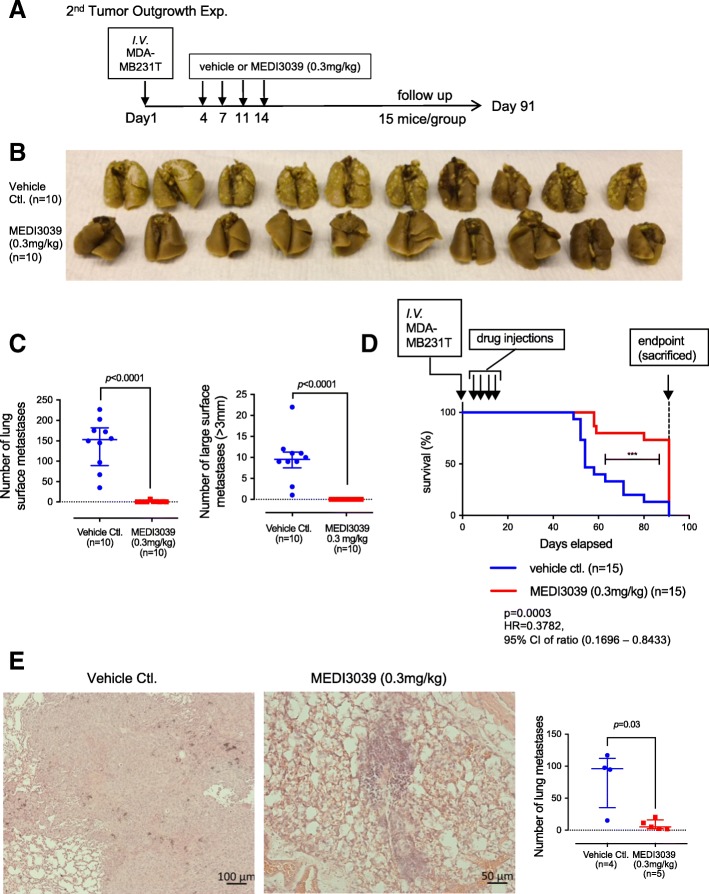
MEDI3039 prevented tumor metastasis outgrowth and extended animal survival in the MB231T experimental pulmonary metastasis model. **a** Design of the experiment. MEDI3039 (0.3 mg/kg) or vehicle was administered twice weekly, for 2 weeks. **b** Mice lung tissue from both vehicle- and MEDI3039-treated groups, fixed with Bouins’ solution. **c** Quantitative analysis of metastasized tumors in the lung. Total numbers of surface metastases (left) and large (> 3 mm) metastases (right) are shown. Data is presented as median with IQR. **d** Survival curve of mice treated with MEDI3039 (0.3 mg/kg) or vehicle. *p* value, HR, and 95% of CI were obtained with log-rank (Mantel–Cox) test, compared with the vehicle control group. **e** Representative images of H&E-stained lung tissue from vehicle- or MEDI3039-treated mouse. Scatter dot graph on the right shows quantitative analysis of microscopic tumors in the lung. Note that the vehicle group has only four samples, while the MEDI3039 group has five samples. One mouse in the vehicle group developed large confluent tumors covering the entire lung, making it difficult to count tumor numbers; therefore, the result is not included in the graph. Data is presented as median with IQR

Next, we examined if MEDI3039 can prevent lung metastasis development in mice in a tail vein experimental pulmonary metastasis model. In the first experiment (Additional file 6), MEDI3039 was tested at two doses (0.3 and 1.0 mg/kg, *n* = 15 each) and compared with the vehicle control (*n* = 15). Beginning 4 days after MB231T cells were injected via tail vein, drug or vehicle was administered twice per week, for 2 weeks for a total of four doses. On day 56, all the mice were euthanized, and the lungs from 10 mice per group were fixed in Bouin’s. The lungs from 5 mice per group were processed for FFPE and examined with H&E staining. MEDI3039 significantly inhibited gross lung metastases at both 0.3 and 1.0 mg/kg doses (*p* = 0.0078 for both, compared with the vehicle control, Additional file 6B, C). There was no statistical difference between 0.3 and 1.0 mg/kg, and no statistical difference in number of large tumors (> 3 mm) among three groups. Analysis of H&E-stained histologic sections revealed a dramatic, statistically significant reduction in the number of metastases in the MEDI3039-treated mice. MEDI3039 significantly inhibited lung metastases at both 0.3 and 1.0 mg/kg doses (*p* = 0.008 for both, compared with the vehicle control, Additional file 6D). There was no statistical difference between 0.3 and 1.0 mg/kg MEDI3039. In the second experiment (Fig. 5), mice were treated with either vehicle control (*n* = 15) or MEDI3039 (0.3 mg/kg, *n* = 15), again administered twice per week, for 2 weeks for a total of four doses (Fig. 5a) and followed up longer, to 91 days. Lungs from ten mice per group were fixed in Bouin’s for gross examination, 5 mice per group were processed for FFPE and examined with H&E staining. Similar to the first experiment (Additional file 6), MEDI3039 (0.3 mg/kg) prevented the development of metastasized tumors in the lung surface (Fig. 5b, c). The median number of surface metastases in the vehicle control group was 153 (IQR = 89.5–182), whereas that of the MEDI3039-treated group was 0 (IQR = 0–1). MEDI3039 significantly (*p* = 0.0003) extended the animal survival. The hazard ratio of 0.3 mg/kg MEDI3039 was 0.3782 compared with the vehicle control, with 95% CI ratio of 0.1696–0.8433; median survival of control group was ~ 50 days, while that of MEDI3039-treated animals was not reached at 91 days when the animals were sacrificed (Fig. 5d). H&E analysis from 5 mice per group also confirmed that metastases in histologic sections were significantly (*p* = 0.03) inhibited by MEDI3039 (Fig. 5e). The median number of lung metastases in the vehicle control group was 96.10 (IQR = 35–112.2), whereas that of the MEDI3039-treated group was 5.2 (IQR = 2.3–16).

### MEDI3039 inhibited growth of established experimental metastases and extended animal survival

**Fig. 6:**
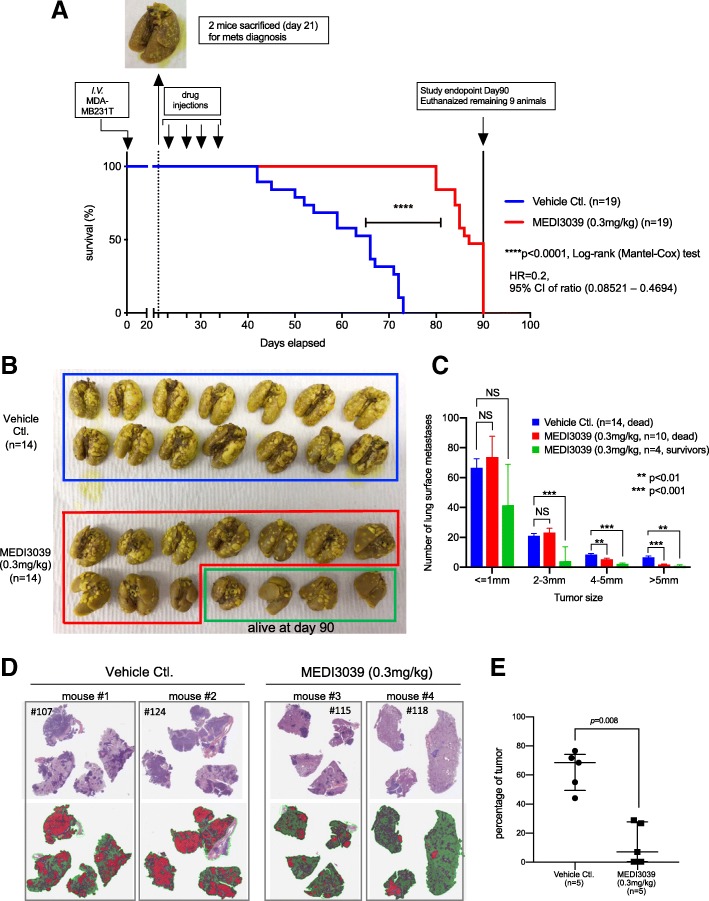
MEDI3039 inhibited established lung metastases and extended animal survival in the MB231T lung metastasis model. **a** Survival curve of mice treated with MEDI3039 (0.3 mg/kg) or vehicle. *p* value, HR, and 95% of CI were obtained with log-rank (Mantel–Cox) test, compared with the vehicle control group. The lung from one of two sentinel mice sacrificed is shown in the inset above the graph demonstrating grossly visible established lung metastases at day 21. **b** Mice lung tissue from both vehicle- and MEDI3039-treated groups, fixed with Bouins’ solution. **c** Quantitative analysis of metastasized tumors in the lung. The number of tumors was classified according to their size. Data is presented as mean ± SEM, and Student’s *t* test was used for the statistical analysis. The red bars represent MEDI3039-treated mice that were sacrificed prior to the end of the experiment. The green bars represent data from 4 mice still alive at the end of the experiment. **d** Two representative H&E-stained lung samples per vehicle- or MEDI3039-treated group. Top: H&E-stained lung tissue. Bottom: red-colored area indicates tumor region, green-colored area indicates non-cancerous region. Areas circled with light dotted green line indicate the diaphragm and lymph nodes excluded from analysis. **e** Quantitative analysis of microscopic tumors in the lung. Data is presented as median area of lung involved with tumor with IQR. Note that all mice in vehicle groups were dead or euthanized on day 72 or 73, while all 5 mice in MEDI3039 were alive and euthanized at day 90

In our third in vivo model system, we examined if MEDI3039 improves outcomes in an established experimental lung metastasis model (Fig. 6). Mice were injected with MDA-MB231T cells via the tail vein. Three weeks later, established lung tumors were documented in 2 mice that were sacrificed at day 20 (representative picture in Fig. 6a). Subsequently, MEDI3039 was administered four times on days 22, 27, 30, and 34, and animals (*n* = 19 in the vehicle group, *n* = 19 in the MEDI3039 group) were followed up to 90 days. By day 73, all the mice in the vehicle group died, whereas the MEDI3039-treated group showed extended survival (Fig. 6a). On day 90, 9/19 mice (47%) in the MEDI3039-treated group were alive. The hazard ratio of MEDI3039-treated group was 0.2 compared with the vehicle control, with 95% CI ratio of 0.08521–0.4694; median survival of vehicle-treated mice was 66 days, and that of MEDI3039 group was 87 days. Analysis of Bouin’s fixed lung tissues (14 mice per group) revealed that growth of lung surface metastases was significantly inhibited by MEDI3039 treatment (Fig. 6b, c). Lungs from the vehicle control group (*n* = 14), MEDI3039-treated and died before day 90 (*n* = 10), and MEDI3039-treated and alive on day 90 (*n* = 4) were indicated with blue, green, and red, respectively (Fig. 6b, c). The group of MEDI3039-treated and dead before day 90 showed significantly fewer surface tumors of 4–5 mm (*p* < 0.01) and > 5 mm (*p* < 0.001) compared with the vehicle control. The group of MEDI3039-treated and alive on day 90 showed significantly fewer surface tumors of 2–3 mm (*p* < 0.001), 4–5 mm (*p* < 0.001), or > 5 mm (*p* < 0.01) compared with the vehicle control. There was no difference in the numbers of small tumors (< 1 mm) among all three groups. H&E-stained lung tissue sections (5 mice per group) confirmed a strong inhibitory effect of MEDI3039 on growth of metastases (*p* < 0.01) (Fig. 6d, e). Tumors in the control animals frequently merged together preventing accurate counting based on the number of metastases. Therefore, the percentage of the lung area involved with metastases (red surface in Fig. 6d) was quantified as outlined in the “Methods and materials” section. The median percentage of the lungs involved with metastases in the vehicle control group was 68.46 (IQR = 49.52–74.2), whereas that of the MEDI3039-treated group was 7.03 (IQR = 0.374–27.87).

## Discussion

Patients with TNBC have a poor prognosis relative to patients with other breast cancer subtypes, and no clinically validated molecularly targeted therapies that improve survival have been identified [2]. Targeting death receptor-mediated apoptosis is an attractive strategy for the treatment of various cancers including TNBC. However, clinical trials with TRAIL receptor agonists have been disappointing (reviewed in [13, 31]). Among the reasons cited for the failure of TRAIL agonists have been key drug characteristics, including potency and half-life. In this study, we tested MEDI3039, a new multivalent DR5 high potency agonist. MEDI3039 killed multiple breast cancer cell lines in vitro, but the sensitivity varied among different subtypes. Sensitivity was particularly highest in basal B TNBC, similar to what we had previously observed with GST-TRAIL [9]; however, MEDI3039 was at least two orders of magnitude more potent compared with GST-TRAIL. Subsequent experiments confirmed that MEDI3039’s effect is dependent on DR5, not DR4, and on caspase activity. MEDI3039 significantly inhibited tumor growth in an MFP model and two lung metastasis models. MEDI3039 extended animal survival in all models. Our study credentials MEDI3039 as a potent DR5 agonist in breast cancer cells, and as especially active in TNBC.

In addition to issues of potency, another possible reason for the failure of Apo2L/TRAIL in clinical trials is the short half-life observed in patients (30–60 min) which limited the exposure of the tumors to therapeutic levels of the drug [14]. The half-life or Apo2L/TRAIL in non-human primates was similar to that in human studies [14, 32]. MEDI3039 has a significantly longer half-life than Apo2L/TRAIL. In mice, the half-life is 15.4 h, and in cynomolgus monkeys, preliminary data suggest a half-life of 31 h [33], both significantly longer than the 20–30 min for TRAIL [32]. Given the Cmax achieved in mice (~ 1.5 μM) and the longer half-life, this suggests that the levels of MEDI3039 could remain above the IC_50_ that was determined for many of the cell lines for at least a week or more (depending ultimately on the PK in humans and the maximum tolerated dose). This would also allow for weekly or even longer intervals between drug doses which would be of significant benefit for the patients’ quality of life. MEDI3039, a DR5 agonist, may not require the prolonged target saturation that traditional antagonistic biologics require. However, we anticipate that sufficient exposure to saturate any normal target may still be required to maximize drug exposure in the tumor.

While MEDI3039 is more potent than GST-TRAIL (Fig. 3a, b; Additional file 3), the relative sensitivity to the drugs is highly correlated (Fig. 3c). The basal B cell TNBC lines were all sensitive to MEDI3039 and GST-TRAIL, and this is consistent with our and other previous reports (reviewed in [34]). Among the ER+, HER2+, and basal A TNBC cell lines, there were some that were sensitive to MEDI3039 and GST-TRAIL, and others that were resistant at the highest concentrations tested (1000 pM for MEDI3039 and 10,000 pM for GST-TRAIL). In contrast to these results, we had previously reported that the ER+, HER2+, and basal A TNBC did not reach an IC_50_ in response to GST-TRAIL [9]. This difference may be attributed to different duration of the treatment; we treated the cells for 16–24 h in the previous study, while we treated cells for 72 h in this study. This suggests that the relative resistance we saw with short treatments in some cells can be overcome if the cells are exposed to the drugs for longer times. The longer half-life of MEDI3039 seen in mice and cynomolgus monkeys potentially would allow for longer duration of therapeutic concentrations and thus could increase the responses in patients.

The underlying mechanisms that make the basal B TNBC subtype of breast cancer cell lines the most sensitive to TRAIL ligands remain enigmatic. Non-malignant HFF cells were MEDI3039 resistant (Additional file 1C), similar to the previous report that HFF cells are TRAIL-resistant [35]. Mesenchymal markers, such as vimentin, moesin, and receptor tyrosine kinase Axl, are highly expressed in TNBC basal B subtype of breast cancer cells (Additional file 2, [9, 36]), and vimentin and Axl proteins were expressed in a subset of human TNBC tumors from patients [36]. This suggests that vimentin and Axl are useful to identify basal B TNBC patients who would be most likely to benefit from DR5 agonist therapies [36]. These genes, however, do not determine their TRAIL sensitivity [9, 36]. A few non-TNBC cell lines, such as HCC1500, ZR751, and AU565, do not express vimentin nor Axl, yet still sensitive to MEDI3039 (Fig. 3a and Additional files 2 and 3). Our data indicate that patients with basal-B-like TNBC are most likely to benefit from MEDI3039. Further studies to identify predictive biomarkers of sensitivity to MEDI3039 that mechanistically regulate the TRAIL pathway will further increase the likelihood of its successful application in the clinic.

Using a TNBC cell line in both primary tumor growth and experimental metastasis models, we demonstrate encouraging in vivo on-target activity for MEDI3039. In the MFP tumor model, a single dose of MEDI3039 induced activation of caspases (as measured by cleavage of caspase 3), decreased proliferation (measured by Ki67), and caused tumor regression (Fig. 4 and Additional file 4). At the higher doses used in Fig. 4 (0.1 and 0.3 mg/kg), the majority of the tumors remained regressed until the drug injections finished (after 5 weekly doses). While most tumors recurred, there was one mouse in each of the 0.1 and 0.3 mg/kg cohorts that remained tumor free for the duration of the experiment (180 days). With increasing doses of MEDI3039, there was increased median survival that reached significance in the 0.3 mg/kg cohort. Multiple studies reinforce that DR5 targeting strategy has potential in patients with TNBC. For example, lexatumumab (an anti-DR5 agonist antibody) delayed tumor growth in MB231 xenografts as monotherapy or in combination with sorafenib, a multi-kinase inhibitor [37]. Apo2L/TRAIL and AMG655 (an anti-DR5 agonist antibody) showed anti-tumor activity in TNBC xenografts, particularly when combined [38]. Also, tigatuzumab (CS-2008, a humanized DR5 antibody) showed an anti-tumor effect in vitro against basal-like breast cancer, and a clinical trial with this antibody showed signs of efficacy in a subset of patients with TNBC [39]. Our data with MEDI3039 are consistent with these results and suggests that MEDI3039 has activity against established TNBC tumors.

MEDI3039 also showed an anti-metastatic activity. In vivo, MEDI3039 prevented the outgrowth of experimental metastases and prolonged survival of the mice when treatment was initiated 4 days after tumor injection (Fig. 5, and Additional file 6). Previously published results with lexatumumab (an anti-DR5 agonist antibody) in a spontaneous orthotopic metastasis model using the MDA-MB231 cells demonstrated that this DR5 agonist also could reduce metastases to the lungs [40]. Interestingly, some data suggest that TRAIL agonists alone or in combination with other agents can target putative cancer tumor-initiating cells which may be responsible for metastatic outgrowth [41, 42]. Most importantly, our data from an experimental metastasis model conducted in a treatment setting confirmed that MEDI3039 has activity on established lung metastases of breast cancer and extends survival (Fig. 6). Mice in the vehicle control group died by day 73; however, 47% (9/19) of mice in the MEDI3039-treated group were alive at day 90. The hazard ratio of the MEDI3039-treated group compared with the non-treated cohort was 0.2. The established metastasis model would be most relevant to an anticipated phase II trial in the metastatic setting.

MEDI3039 showed strong anti-tumor potency; however, most of tumors recurred and the animals eventually died in both the orthotopic and metastasis models. This limitation of the data is probably pertinent to all cancer drugs extant. TRAIL sensitivity is shown to vary even within a single clone of cells, due to varying levels or states of proteins regulating death receptor-mediated apoptosis [43], reviewed in [44, 45]. Therefore, complete eradication of tumors is challenging, as only a maximum of 4–5 doses of drug treatment were administered in our models. The recurrences are likely due to the failure to fully eradicate the tumors with limited doses of the drug. In the clinical setting with human patients, we envision first testing the drug in the metastatic setting where treatment would continue indefinitely as long as the tumor was responding and the patient tolerating the drug. If activity is seen there, the drug might be tested in the neoadjuvant or adjuvant setting where the patient would have only micro-metastases, and again the length of treatment would likely be considerably longer. Also, combination therapeutic regimens need to be investigated. Ongoing work is investigating mechanisms of drug resistance.

Together, our data demonstrate that MEDI3039 has anti-tumor activity in human breast cancer that warrants further investigation in clinical trials.

## Conclusions

MEDI3039 is a highly potent TRAIL receptor agonist in breast cancer cells and has potential as a cancer drug in breast cancer patients, especially those with basal B TNBC.

## Additional files

Additional file 1: MEDI3039 induces cell death in TNBC cell lines. (A) Western blot showing time-dependent effect of MEDI3039 (1pM) on MB231 cells. One of two experiments showing similar results. (B) Comparison of 16 h and 72 h treatment of MEDI3039 in MB231 and MB468 cells by MTS assays. Data is presented as mean +/− SD, ****p < 0.0001, two-way ANOVA. The data is one of two experiments in each cell line. (C) Left: comparison of cell viability (MTS) assay of MB231 and HFF treated with MEDI3039. Data is presented as mean +/− SEM of multiple experiments. ****p < 0.0001, two-way ANOVA. Right: Western blot showing expression of DR4, DR5, Axl, Vimentin in MB231 and HFF cells. HSC70 was used as a loading control. The numbers shown indicate relative band intensity of DR4 and DR5 normalized to HSC70. (D) Live/Dead monitoring of cells treated with MEDI3039 or GST-TRAIL. The results of MB231 cells are at 24 h time point, MB468 and MCF7 cells were at 48 h time point, T47D were at 72 h time point. All data is presented as mean +/− SEM of 3 independent experiments. (E) Time-dependent effect of MEDI3039/TRAIL on cell death. Note that MB468 cells showed time-dependent increase of cell death measured by fluorescence, whereas MCF7 and T47D cells showed it only little or no increase in dead cells compared to untreated controls. All the data is shown as mean+/− SEM of 3 independent experiments. MB231 cells data is not shown as the peak fluorescence was reached at 24 h as shown in (D). (PDF 550 kb)

Additional file 2: Expression of different markers in various breast cancer cell lines. Western blot showing different proteins expressed in various subtypes of 15 breast cancer cell lines. (PDF 1167 kb)

Additional file 3: IC_50_ of MEDI3039 and GST-TRAIL in multiple breast cancer cell lines with different subtypes. N.D. = not determined. (PDF 21 kb)

Additional file 4 First MEDI3039 experiment in MFP model. This experiment was performed prior to dose-response experiment shown in Fig. 4. (A) Design of the experiment. 15 mice were treated with vehicle control or MEDI3039 (0.3 mg/kg), each group. Drugs were administered once a week, for 2 weeks. 5 mice/each group were sacrificed for histology analysis (B) and other 10 mice/group were followed up for tumor growth and survival. (B) Immunohistochemistry analysis of tumor samples from MEDI3039 (0.3 mg/kg) or vehicle-injected mice. Samples were stained with either Ki67 and DAPI or CC3 (cleaved caspase 3) and DAPI. Bar = 100 μm. (C) Quantitative analysis of signal intensity of Ki67 and CC3, both normalized with DAPI. Data is shown as median with IQR. Numbers of mice examined was 5 per each group. *p* value was obtained by Mann–Whitney test. (D) Tumor growth curve in each treatment group. (E) Survival curve of mice treated with vehicle or MEDI3039 (0.3 mg/kg). All 10 mice in the vehicle control group developed tumors more than 20 mm in diameter and needed to be sacrificed on Day 35. Median survival in MEDI3039 (0.3 mg/kg) group was 72 days. *p* value, HR, 95% of CI were obtained with Log-rank (Mantel-Cox) test, compared with the vehicle control group. (PDF 313 kb)

Additional file 5: Histology analysis of extra MFP tumor developed in later stage in a mouse treated with MEDI3039. (A) A mouse developed extra MFP tumor after MEDI3039 treatment. The picture was taken on Day 145 before euthanizing and tumor collection. (B) Immunohistochemistry analysis of the tumor with IgG (negative control), and anti-human mitochondrial antibody. (PDF 210 kb)

Additional file 6: MEDI3039 inhibited tumor metastases and extended animal survival in MB231T lung metastasis model. This experiment was performed prior to the 2nd experiment shown in Fig. 5, to examine the dose-dependent effect of MEDI3039 on metastasis formation. (A) Design of the experiment. MEDI3039 (0.3, 1.0 mg/kg) or vehicle was administered twice weekly, for 2 weeks. (B) Mice lung tissue from each group, fixed with Bouins’ solution. (C) Total numbers of surface metastases (left) and large metastases (> 3 mm) tumor (right) are shown. Data is presented as median with IQR. One-way ANOVA was used to compare statistical significance between different groups. (D) Representative images of H&E stained lung tissue from vehicle or MEDI3039-treated mouse. Microscopic metastasis is indicated with black dotted circle in the image (Vehicle Ctl.). The graph on right shows quantitative analysis of microscopic tumors in lung. Data is presented as median with IQR. *p* value was obtained by Mann–Whitney test . (PDF 237 kb)

## Abbreviations

DR: Death receptor
EC_50_: The half maximal effective concentration
ER: Estrogen receptor
GST-TRAIL: Glutathione synthetase transferase-tagged tumor necrosis factor-related apoptosis-inducing ligand
HER2: Human epidermal growth factor receptor 2
IC_50_: The half maximal inhibitory concentration
IQR: Interquartile range
MB231: MDA-MB231
MB468: MDA-MB468
MFP: Mammary fat pad
MTS: 3-(4,5-Dimethylthiazol-2-yl)-5-(3-carboxymethoxyphenyl)-2-(4-sulfophenyl)-2H-tetrazolium, inner salt
PR: Progesterone receptor
SEM: Standard error of the mean
siRNA: Small interfering ribonucleic acid
TNBC: Triple-negative breast cancer
TNF: Tumor necrosis factor
Z-VAD-FMK: Carbobenzoxy-valyl-alanyl-aspartyl-[O-methyl]-fluoromethylketone
