# Supplementary figures and images for: MEDI3039, a novel highly potent tumor necrosis factor (TNF)-related apoptosis-inducing ligand (TRAIL) receptor 2 agonist, causes regression of orthotopic tumors and inhibits outgrowth of metastatic triple-negative breast cancer
Source: Breast Cancer Res. 2019 Feb 18;21:27. doi: 10.1186/s13058-019-1116-1 (PMC6380056; doi:10.1186/s13058-019-1116-1)

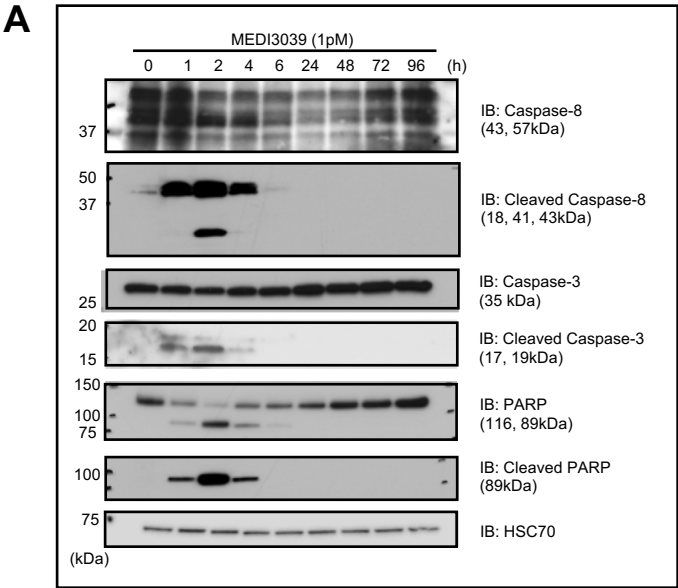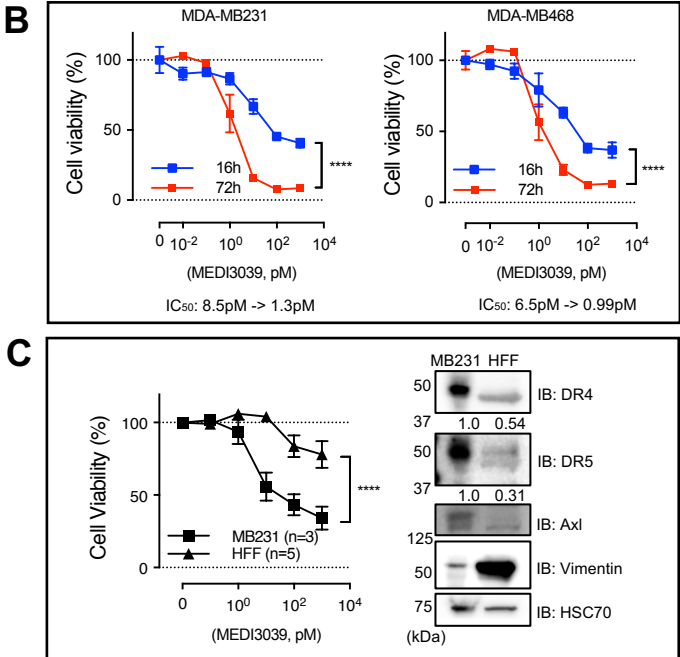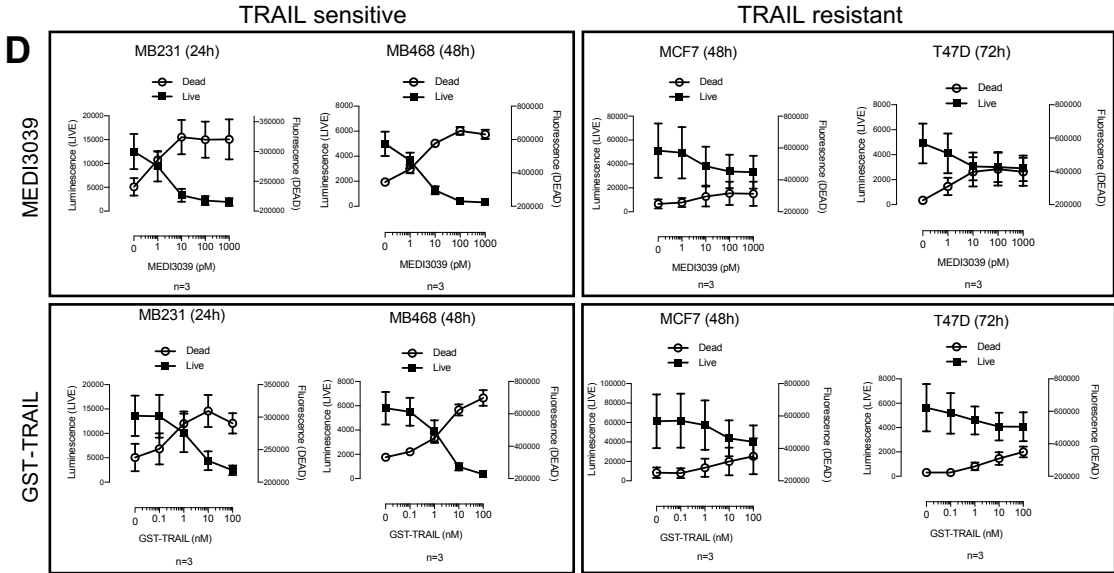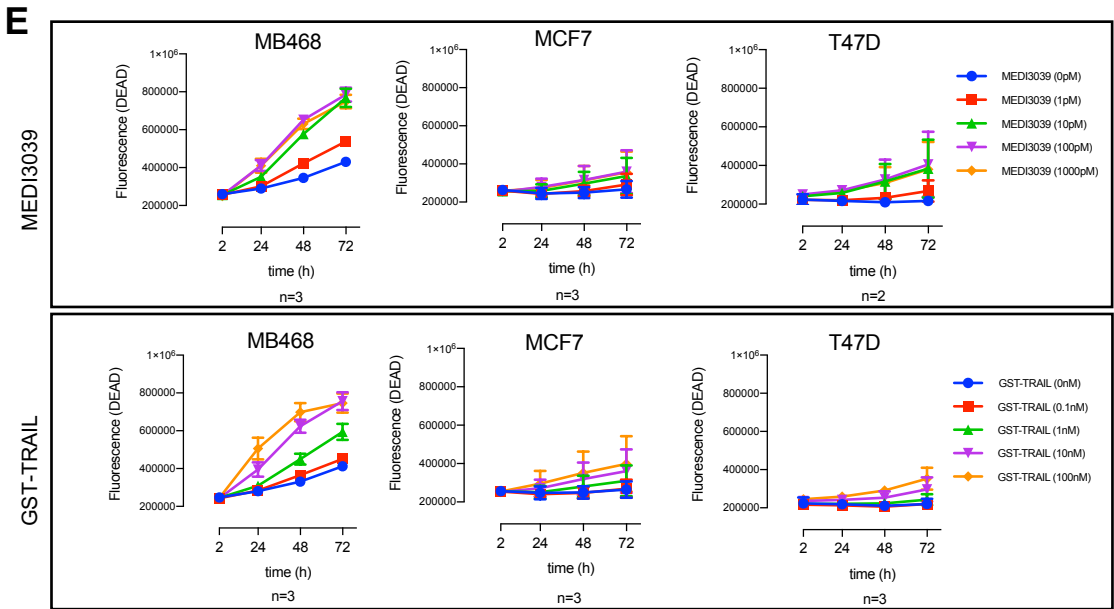

Supplement: Supplementary file 1 — MEDI3039 induces cell death in TNBC cell lines. (A) Western blot showing time-dependent effect of MEDI3039 (1pM) on MB231 cells. One of two experiments showing similar results. (B) Comparison of 16 h and 72 h treatment of MEDI3039 in MB231 and MB468 cells by MTS assays. Data is presented as mean +/− SD, ****p < 0.0001, two-way ANOVA. The data is one of two experiments in each cell line. (C) Left: comparison of cell viability (MTS) assay of MB231 and HFF treated with MEDI3039. Data is presented as mean +/− SEM of multiple experiments. ****p < 0.0001, two-way ANOVA. Right: Western blot showing expression of DR4, DR5, Axl, Vimentin in MB231 and HFF cells. HSC70 was used as a loading control. The numbers shown indicate relative band intensity of DR4 and DR5 normalized to HSC70. (D) Live/Dead monitoring of cells treated with MEDI3039 or GST-TRAIL. The results of MB231 cells are at 24 h time point, MB468 and MCF7 cells were at 48 h time point, T47D were at 72 h time point. All data is presented as mean +/− SEM of 3 independent experiments. (E) Time-dependent effect of MEDI3039/TRAIL on cell death. Note that MB468 cells showed time-dependent increase of cell death measured by fluorescence, whereas MCF7 and T47D cells showed it only little or no increase in dead cells compared to untreated controls. All the data is shown as mean+/− SEM of 3 independent experiments. MB231 cells data is not shown as the peak fluorescence was reached at 24 h as shown in (D). (PDF 550 kb) [file 13058_2019_1116_MOESM1_ESM.pdf]

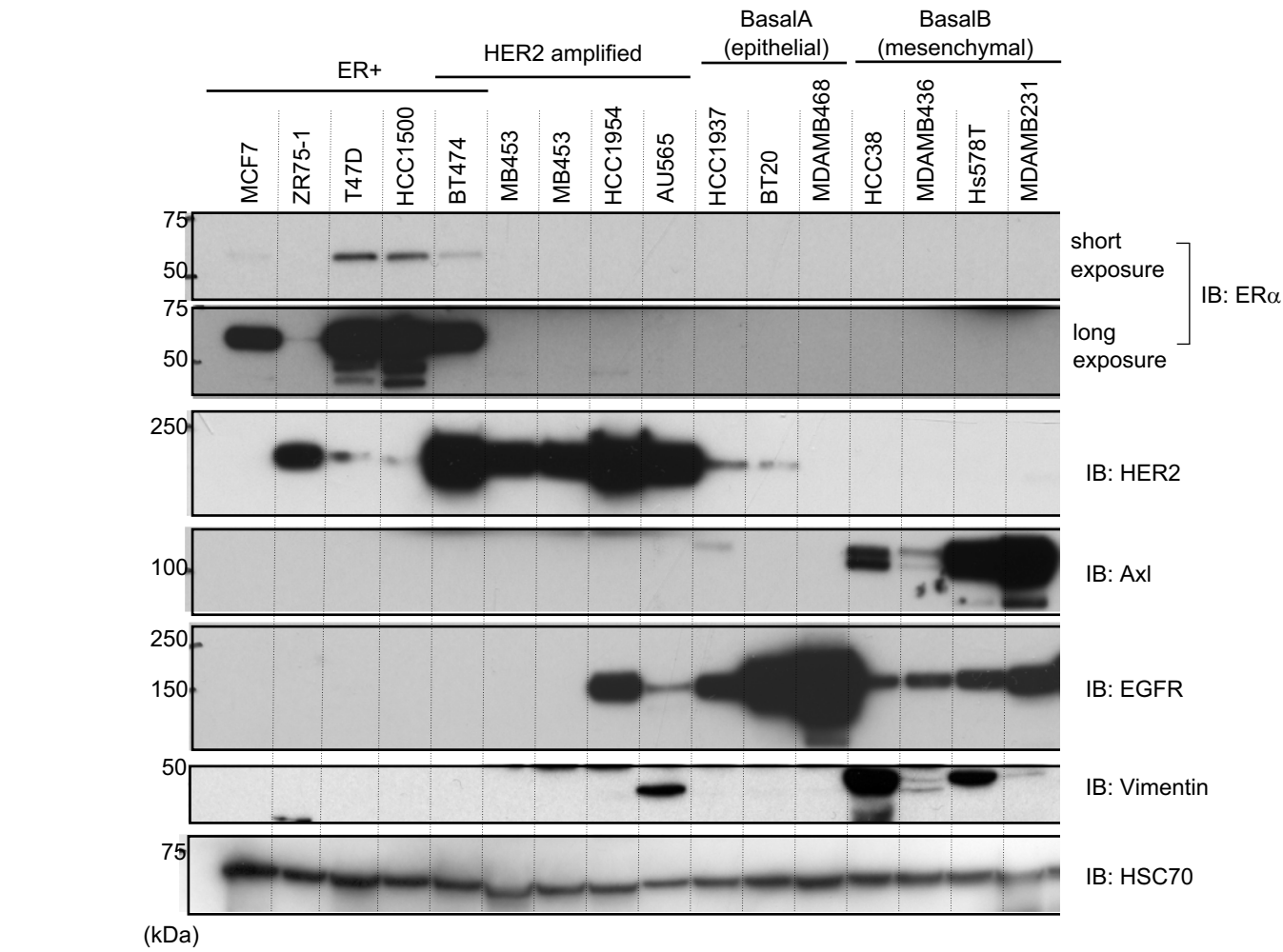

Supplement: Supplementary file 2 — Expression of different markers in various breast cancer cell lines. Western blot showing different proteins expressed in various subtypes of 15 breast cancer cell lines. (PDF 1167 kb) [file 13058_2019_1116_MOESM2_ESM.pdf]

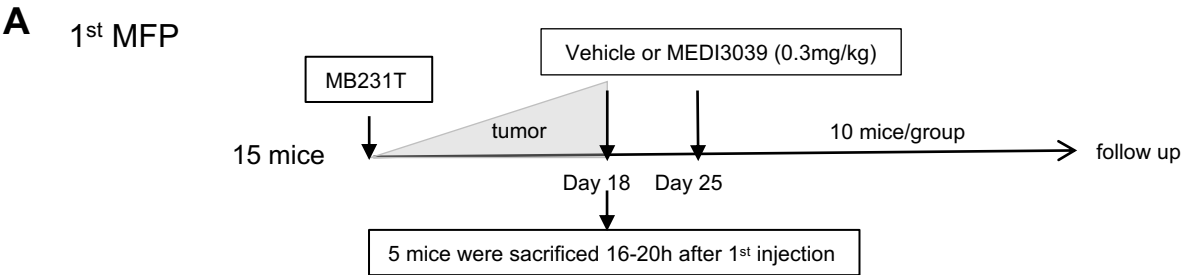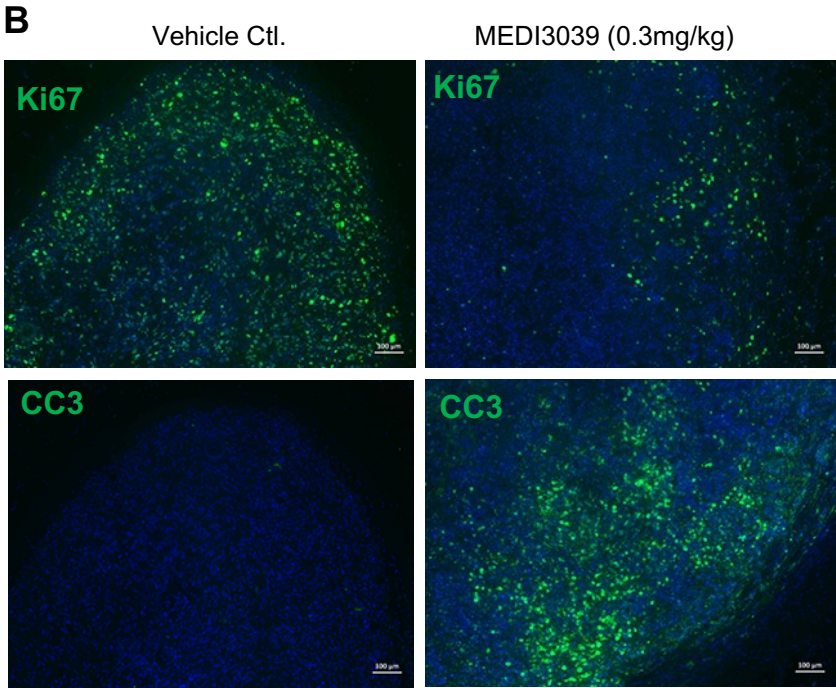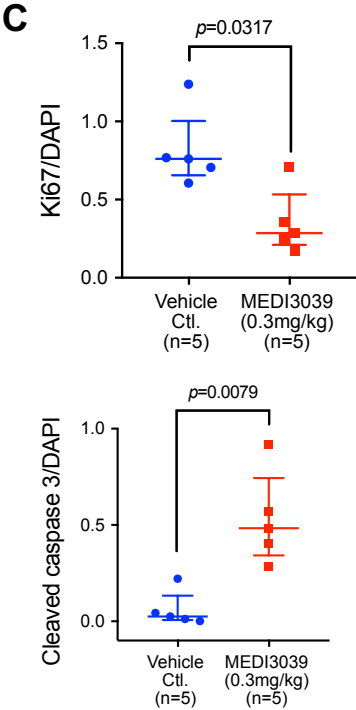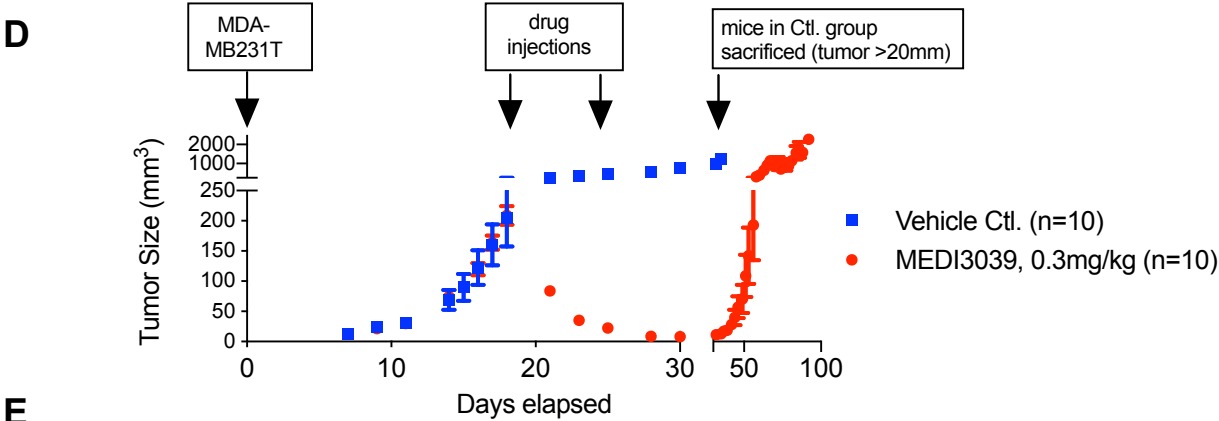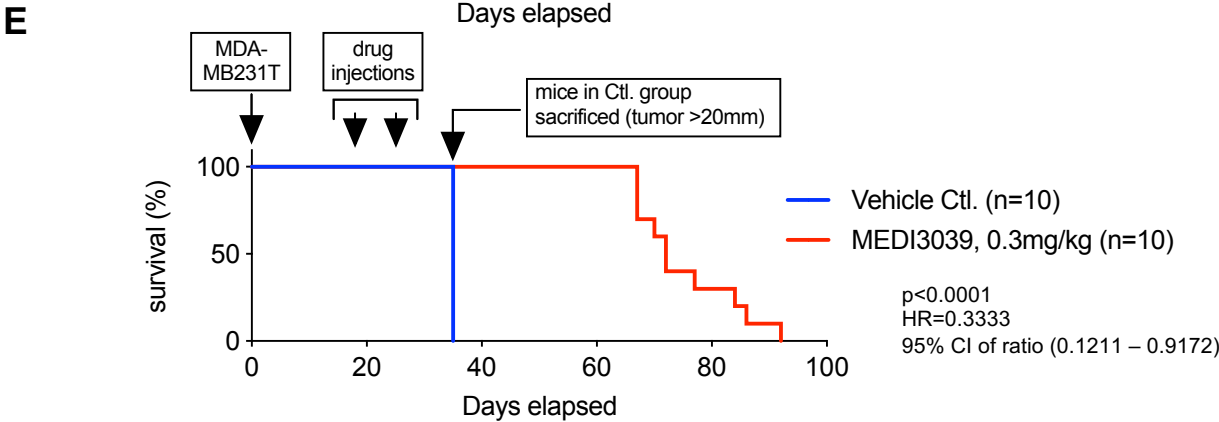

Supplement: Supplementary file 4 — First MEDI3039 experiment in MFP model. This experiment was performed prior to dose-response experiment shown in Fig. 4. (A) Design of the experiment. 15 mice were treated with vehicle control or MEDI3039 (0.3 mg/kg), each group. Drugs were administered once a week, for 2 weeks. 5 mice/each group were sacrificed for histology analysis (B) and other 10 mice/group were followed up for tumor growth and survival. (B) Immunohistochemistry analysis of tumor samples from MEDI3039 (0.3 mg/kg) or vehicle-injected mice. Samples were stained with either Ki67 and DAPI or CC3 (cleaved caspase 3) and DAPI. Bar = 100 μm. (C) Quantitative analysis of signal intensity of Ki67 and CC3, both normalized with DAPI. Data is shown as median with IQR. Numbers of mice examined was 5 per each group. p value was obtained by Mann–Whitney test. (D) Tumor growth curve in each treatment group. (E) Survival curve of mice treated with vehicle or MEDI3039 (0.3 mg/kg). All 10 mice in the vehicle control group developed tumors more than 20 mm in diameter and needed to be sacrificed on Day 35. Median survival in MEDI3039 (0.3 mg/kg) group was 72 days. p value, HR, 95% of CI were obtained with Log-rank (Mantel-Cox) test, compared with the vehicle control group. (PDF 313 kb) [file 13058_2019_1116_MOESM4_ESM.pdf]

**A**

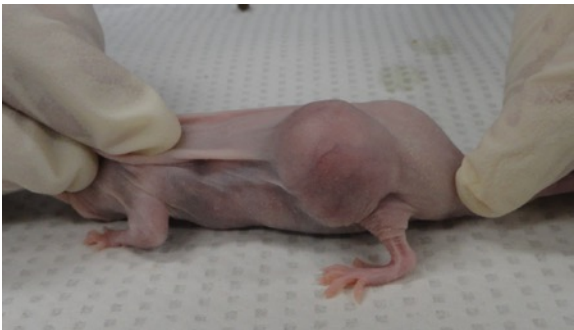

**B**

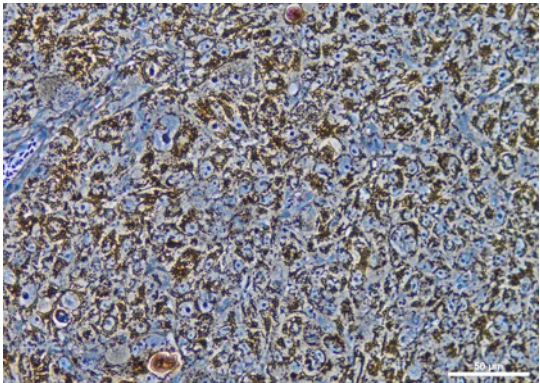

Anti-human mitochondria  
antibody [MTC02], ab79479

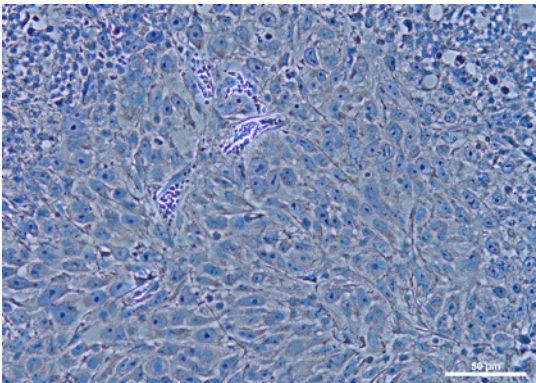

IgG Ctl.

Supplement: Supplementary file 5 — Histology analysis of extra MFP tumor developed in later stage in a mouse treated with MEDI3039. (A) A mouse developed extra MFP tumor after MEDI3039 treatment. The picture was taken on Day 145 before euthanizing and tumor collection. (B) Immunohistochemistry analysis of the tumor with IgG (negative control), and anti-human mitochondrial antibody. (PDF 210 kb) [file 13058_2019_1116_MOESM5_ESM.pdf]

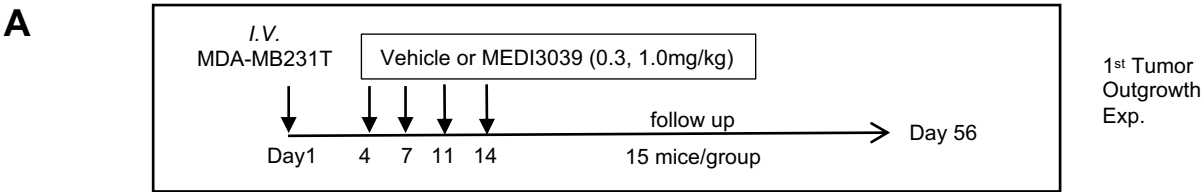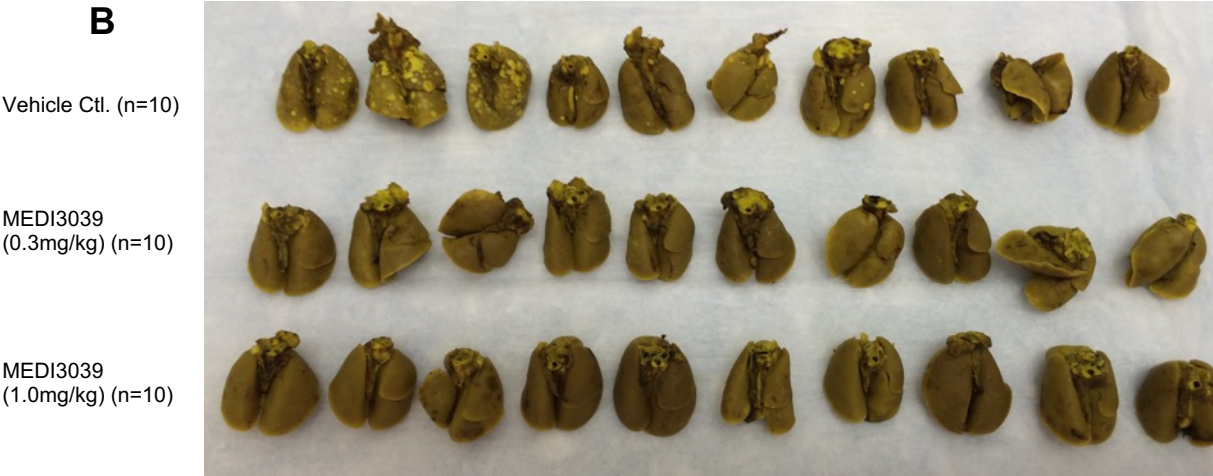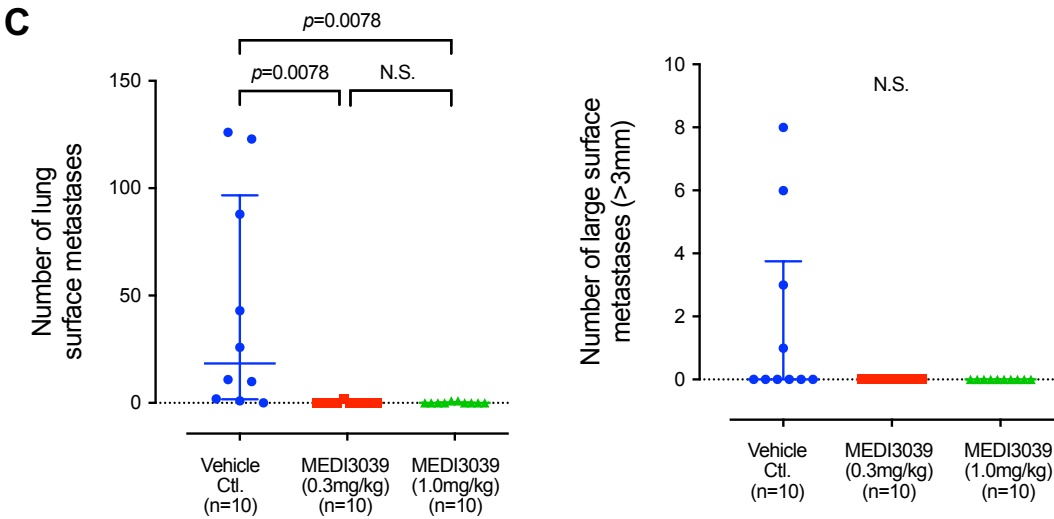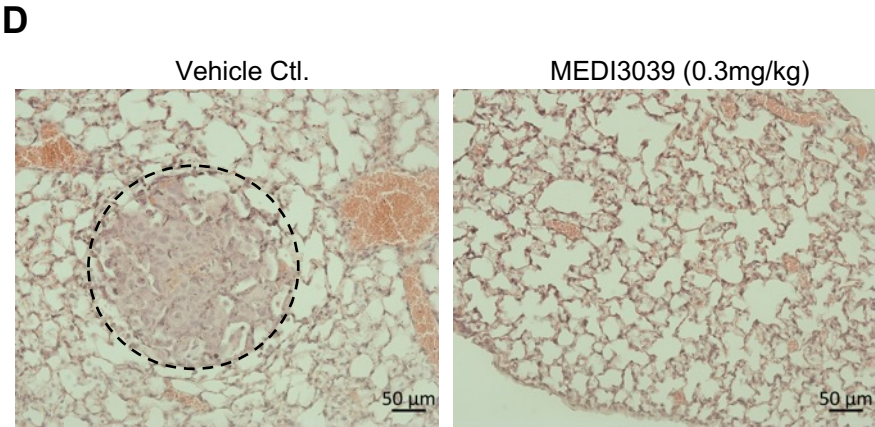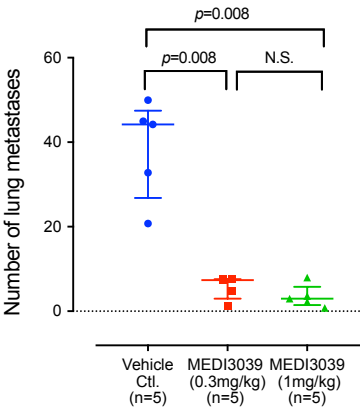

Supplement: Supplementary file 6 — MEDI3039 inhibited tumor metastases and extended animal survival in MB231T lung metastasis model. This experiment was performed prior to the 2nd experiment shown in Fig. 5, to examine the dose-dependent effect of MEDI3039 on metastasis formation. (A) Design of the experiment. MEDI3039 (0.3, 1.0 mg/kg) or vehicle was administered twice weekly, for 2 weeks. (B) Mice lung tissue from each group, fixed with Bouins’ solution. (C) Total numbers of surface metastases (left) and large metastases (> 3 mm) tumor (right) are shown. Data is presented as median with IQR. One-way ANOVA was used to compare statistical significance between different groups. (D) Representative images of H&E stained lung tissue from vehicle or MEDI3039-treated mouse. Microscopic metastasis is indicated with black dotted circle in the image (Vehicle Ctl.). The graph on right shows quantitative analysis of microscopic tumors in lung. Data is presented as median with IQR. p value was obtained by Mann–Whitney test . (PDF 237 kb) [file 13058_2019_1116_MOESM6_ESM.pdf]
