# Supplementary material for: MEDI3039, a novel highly potent tumor necrosis factor (TNF)-related apoptosis-inducing ligand (TRAIL) receptor 2 agonist, causes regression of orthotopic tumors and inhibits outgrowth of metastatic triple-negative breast cancer
Source: Breast Cancer Res. 2019 Feb 18;21:27. doi: 10.1186/s13058-019-1116-1 (PMC6380056; doi:10.1186/s13058-019-1116-1)
Supplement: Supplementary file 3 — IC50 of MEDI3039 and GST-TRAIL in multiple breast cancer cell lines with different subtypes. N.D. = not determined. (PDF 21 kb) [file 13058_2019_1116_MOESM3_ESM.pdf]

Group	cell line	GST-TRAIL (IC50, pM)	MEDI3039 (IC50, pM)	fold difference
ER+	T47D	>100,000.00	> 1,000.00	N.D.
	MCF7	7,422.00	8.92	832.25
	HCC1500	2,410.00	4.22	571.63
	ZR75-1	2,184.00	0.61	3563.39
	BT474	>100,000.00	> 1,000.00	N.D.
HER2 amplified	MB453	15,038.00	245.80	61.18
	SKBR3-ATCC	>100,000.00	>1,000.00	N.D.
	HCC1954	1,734.00	6.97	248.78
	AU565	1,757.00	2.90	606.91
TNBC BasalA	HCC1599	>100,000.00	>1,000.00	N.D.
	HCC1937	1,990.00	3.87	514.61
	MB468	1,268.00	7.83	161.92
	HCC1187	988.70	0.90	1093.21
	BT20	464.40	0.38	1235.76
TNBC BasalB	MB231	623.90	4.71	132.46
	Hs578T	447.80	1.84	242.97
	MB436	80.20	0.15	532.89
	BT549	66.44	0.22	308.74
	HCC38	6.73	0.06	115.71
